# Characterizing the Epidemiology of the 2009 Influenza A/H1N1 Pandemic in Mexico

**DOI:** 10.1371/journal.pmed.1000436

**Published:** 2011-05-24

**Authors:** Gerardo Chowell, Santiago Echevarría-Zuno, Cécile Viboud, Lone Simonsen, James Tamerius, Mark A. Miller, Víctor H. Borja-Aburto

**Affiliations:** 1 Mathematical, Computational & Modeling Sciences Center, School of Human Evolution and Social Change, Arizona State University, Tempe, Arizona, United States of America; 2 Division of Epidemiology and Population Studies, Fogarty International Center, National Institutes of Health, Bethesda, Maryland, United States of America; 3 Dirección de Prestaciones Médicas, Instituto Mexicano del Seguro Social, Mexico City; 4 Department of Global Health, School of Public Health and Health Services, George Washington University, Washington (D.C.), United States of America; 5 School of Geography and Development, University of Arizona, Tucson, Arizona, United States of America; 6 Coordinación de Vigilancia Epidemiológica y Apoyo en Contingencias Instituto Mexicano del Seguro Social, Mier y Pesado 120, México, México; The University of Hong Kong, Hong Kong

## Abstract

**Background:**

Mexico's local and national authorities initiated an intense public health response during the early stages of the 2009 A/H1N1 pandemic. In this study we analyzed the epidemiological patterns of the pandemic during April–December 2009 in Mexico and evaluated the impact of nonmedical interventions, school cycles, and demographic factors on influenza transmission.

**Methods and Findings:**

We used influenza surveillance data compiled by the Mexican Institute for Social Security, representing 40% of the population, to study patterns in influenza-like illness (ILIs) hospitalizations, deaths, and case-fatality rate by pandemic wave and geographical region. We also estimated the reproduction number (R) on the basis of the growth rate of daily cases, and used a transmission model to evaluate the effectiveness of mitigation strategies initiated during the spring pandemic wave. A total of 117,626 ILI cases were identified during April–December 2009, of which 30.6% were tested for influenza, and 23.3% were positive for the influenza A/H1N1 pandemic virus. A three-wave pandemic profile was identified, with an initial wave in April–May (Mexico City area), a second wave in June–July (southeastern states), and a geographically widespread third wave in August–December. The median age of laboratory confirmed ILI cases was ∼18 years overall and increased to ∼31 years during autumn (*p*<0.0001). The case-fatality ratio among ILI cases was 1.2% overall, and highest (5.5%) among people over 60 years. The regional R estimates were 1.8–2.1, 1.6–1.9, and 1.2–1.3 for the spring, summer, and fall waves, respectively. We estimate that the 18-day period of mandatory school closures and other social distancing measures implemented in the greater Mexico City area was associated with a 29%–37% reduction in influenza transmission in spring 2009. In addition, an increase in R was observed in late May and early June in the southeast states, after mandatory school suspension resumed and before summer vacation started. State-specific fall pandemic waves began 2–5 weeks after school reopened for the fall term, coinciding with an age shift in influenza cases.

**Conclusions:**

We documented three spatially heterogeneous waves of the 2009 A/H1N1 pandemic virus in Mexico, which were characterized by a relatively young age distribution of cases. Our study highlights the importance of school cycles on the transmission dynamics of this pandemic influenza strain and suggests that school closure and other mitigation measures could be useful to mitigate future influenza pandemics.

*Please see later in the article for the Editors' Summary*

## Abstract 2

Gerardo Chowell and colleagues address whether school closures and other social distancing strategies were successful in reducing pandemic flu transmission in Mexico by analyzing the age- and state-specific incidence of influenza morbidity and mortality in 32 Mexican states.

## Editors' Summary

**Background:**

From June 2009 to August 2010, the world was officially (according to specific World Health Organization [WHO] criteria—WHO phase 6 pandemic alert) in the grip of an Influenza A pandemic with a new strain of the H1N1 virus. The epidemic in Mexico, which had the second confirmed global case of H1N1 virus was first noted in early April 2009, when reports of respiratory hospitalizations and deaths among 62 young adults in Mexico alerted local health officials to the occurrence of atypical rates of respiratory illness. In line with its inter-institutional National Pandemic Influenza Preparedness and Response Plan, the Ministry of Health cancelled school attendance in the greater Mexico City area on April 24 and expanded these measures to the rest the country three days later. The Ministry of Health then implemented in Mexico City other “social distancing” strategies such as closing cinemas and restaurants and cancelling large public gatherings.

**Why Was This Study Done?:**

School closures and other intense social distancing strategies can be very disruptive to the population, but as yet it is uncertain whether these measures were successful in reducing disease transmission. In addition, there have been no studies concentrating on recurrent pandemic waves in Mexico. So in this study the authors addressed these issues by analyzing the age- and state-specific incidence of influenza morbidity and mortality in 32 Mexican States and quantified the association between local influenza transmission rates, school cycles, and demographic factors.

**What Did the Researchers Do and Find?:**

The researchers used the epidemiological surveillance system of the Mexican Institute for Social Security—a Mexican health system that covers private sector workers and their families, a group representative of the general population, that comprises roughly 40% of the Mexican population (107 million individuals), with a network of 1,099 primary health care units and 259 hospitals nationwide. Then the researchers compiled state- and age-specific time series of incident influenza-like illness and H1N1 influenza cases by day of symptom onset to analyze the geographic dissemination patterns of the pandemic across Mexico and defined three temporally distinct pandemic waves in 2009: spring (April 1–May 20), summer (May 21–August 1), and fall (August 2–December 31). The researchers then applied a mathematical model of influenza transmission to daily case data to assess the effectiveness of mandatory school closures and other social distancing measures implemented during April 24–May 11, in reducing influenza transmission rates.

The Mexican Institute for Social Security reported a total of 117,626 people with influenza-like illness from April 1 to December 31, 2009, of which 36,044 were laboratory tested (30.6%) and 27,440 (23.3%) were confirmed with H1N1 influenza. During this period, 1,370 people with influenza-like illness died of which 585 (1.5 per 100,000) were confirmed to have H1N1 influenza. The median age of people with laboratory confirmed influenza like illness (H1N1) was 18 years overall but increased to 31 years during the autumn wave. The overall case-fatality ratio among people with influenza like illness was 1.2%, but highest (5.5%) among people over 60 years. The researchers found that the 18-day period of mandatory school closures and other social distancing measures implemented in the greater Mexico City area was associated with a substantial (29%–37%) reduction in influenza transmission in spring 2009 but increased in late May and early June in the southeast states, after mandatory school suspension resumed and before summer vacation started. State-specific pandemic waves began 2–5 weeks after school reopened for the fall term, coinciding with an age shift in influenza cases.

**What Do These Findings Mean?:**

These findings show that the age distribution of pandemic influenza morbidity was greater in younger age groups, while the risk of severe disease was skewed towards older age groups, and that there were substantial geographical variation in pandemic patterns across Mexico, in part related to population size. But most importantly, these findings support the effectiveness of early mitigation efforts including mandatory school closures and cancellation of large public gatherings, reinforcing the importance of school cycles in the transmission of pandemic influenza. This analysis increases understanding of the age and transmission patterns of the Mexican 2009 influenza pandemic at various geographic scales, which is crucial for designing more efficient public health interventions against future influenza pandemics.

**Additional Information:**

Please access these Web sites via the online version of this summary at http://dx.doi.org/10.1371/journal.pmed.1000436.

- The World Health Organization provides information about the global response to the 2009 H1N1 pandemic

## Introduction

In late March and early April 2009, reports of respiratory hospitalizations and deaths among young adults in Mexico alerted local health officials to the occurrence of atypical rates of respiratory illness at a time when influenza was not expected to reach epidemic levels [1]–[3]. Infections with novel swine-origin influenza A/H1N1 virus were confirmed in California, (United States), on April 21 [4] and in Mexico on April 23 [5]. The Ministry of Health cancelled educational activities in the greater Mexico City area on April 24 and expanded these measures to the rest of the country on April 27 [6]. Additional social distancing interventions were implemented in the greater Mexico City area, including the closure of movie theaters and restaurants and the cancellation of large public gatherings (Table 1) [6]. Schools reopened on May 11 and remained in session until the scheduled summer vacation period, which began in July 2009. Whether these intense interventions were successful in reducing disease transmission has yet to be evaluated, which is important for the control of future pandemics [7].

**Table 1:** Timeline of events relevant to the detection, control, and school activity periods during the 2009 A/H1N1 influenza pandemic in Mexico.

Dates	Events
April 5–18, 2009	Spring break school vacation period for approximately 34 million students from elementary to university levels.
April 12, 2009	Mexico reports an outbreak of respiratory disease to the Pan-American Health Organization (PAHO)
April 17, 2009	Ministry of Health issues epidemiologic alert
April, 23 2009	The Public Health Agency of Canada confirms cases of novel swine-origin (A/H1N1) influenza virus
April 24–May 11, 2009	Educational activities at all levels are cancelled in the Federal District (Distrito Federal) and the metropolitan area, including the state of Mexico. Movie theaters, restaurants, soccer stadiums, and churches are also temporarily closed in the greater Mexico City metropolitan area
April 27–May 11, 2009	School closures are extended to the rest of the country
July 3, 2009	Summer school vacation period begins
August 10, 2009	Start of the school term for university students
August 24, 2009	Start of the school term for public primary and secondary schools
December 22, 2009	Winter school vacation period begins

Increasing our understanding of the age and transmission patterns of the 2009 A/H1N1 influenza pandemic at various geographic scales is crucial for designing more efficient public health interventions against future influenza pandemics. Spatio-temporal variations in influenza transmission can result from variation in population contact rates linked to school cycles or intervention strategies, as well as the timing of a virus's introduction relative to climatic conditions and prior population immunity (e.g., [8],[9]). While variation in the transmission potential and the timing of the spring waves of the 2009 A/H1N1 pandemic have been reported in several countries (e.g., [10]–[16]), there have been no studies thus far concentrating on recurrent pandemic waves in Mexico, one of the countries affected earliest by the 2009 A/H1N1 influenza pandemic. Here, we analyze the age- and state-specific incidence of influenza morbidity and mortality in 32 Mexican States, on the basis of reports to the Mexican Institute for Social Security (IMSS), a private medical system that covers 40% of the Mexican population. We also quantify the association between local influenza transmission rates, school cycles, and demographic factors.

## Methods

### Epidemiological and Population Data

We relied on the epidemiological surveillance system of IMSS, described in detail by Echevarria-Zuno et al. [17]. IMSS is a tripartite Mexican health system covering workers in the private sector and their families, a group that comprises roughly 40% of the Mexican population (107 million individuals), with a network of 1,099 primary health care units and 259 hospitals nationwide. Overall, the age distribution of the population affiliated with IMSS is representative of the general population of Mexico (chi-square test, *p* = 0.18) (Text S1, figure A) [18]. The male-to-female ratio among the population affiliated with IMSS (47:53) is similar to that of the general population (49:51).

Active surveillance for severe pneumonia started at all IMSS hospitals after a first epidemiological alert was issued on April 17, 2009. On April 28 the surveillance system was expanded to include influenza-like illness (ILI) patients visiting primary health care units and hospitals as well as influenza-related deaths. Patient information was entered into an online surveillance system by hospital or clinic epidemiologists. ILI was defined as a combination of cough, headache, and fever (except for persons over 65 y) with one or more of the following symptoms: sore throat, rhinorrhea, arthralgias, myalgia, prostration, thoracic pain, abdominal pain, nasal congestion, diarrhea, and irritability (for infants only) [17]. Respiratory swabs were obtained for about a third of cases with constant sampling intensity across states, time, and age groups (Text S1, figures B and C and table A). Swabs were tested for A/H1N1 influenza virus by real-time reverse transcription PCR [19] by the Instituto de Diagnóstico y Referencia Epidemiológica (InDRE) until May 25, 2009, after which point samples were analyzed by La Raza, an IMSS laboratory certified by InDRE [17].

We obtained patient age, date of symptom onset, disease outcome (inpatient, outpatient, and death), and reporting state (including 31 states plus the Federal District, which we collectively refer to as “32 states” for simplicity) for ILI and laboratory-confirmed A/H1N1 pandemic influenza cases reported between April 1 and December 31, 2009. We also obtained population data by state and age group for all persons affiliated with IMSS in 2009 to calculate incidence rates.

### Spatial Distribution of Pandemic Waves

**Figure 1:**
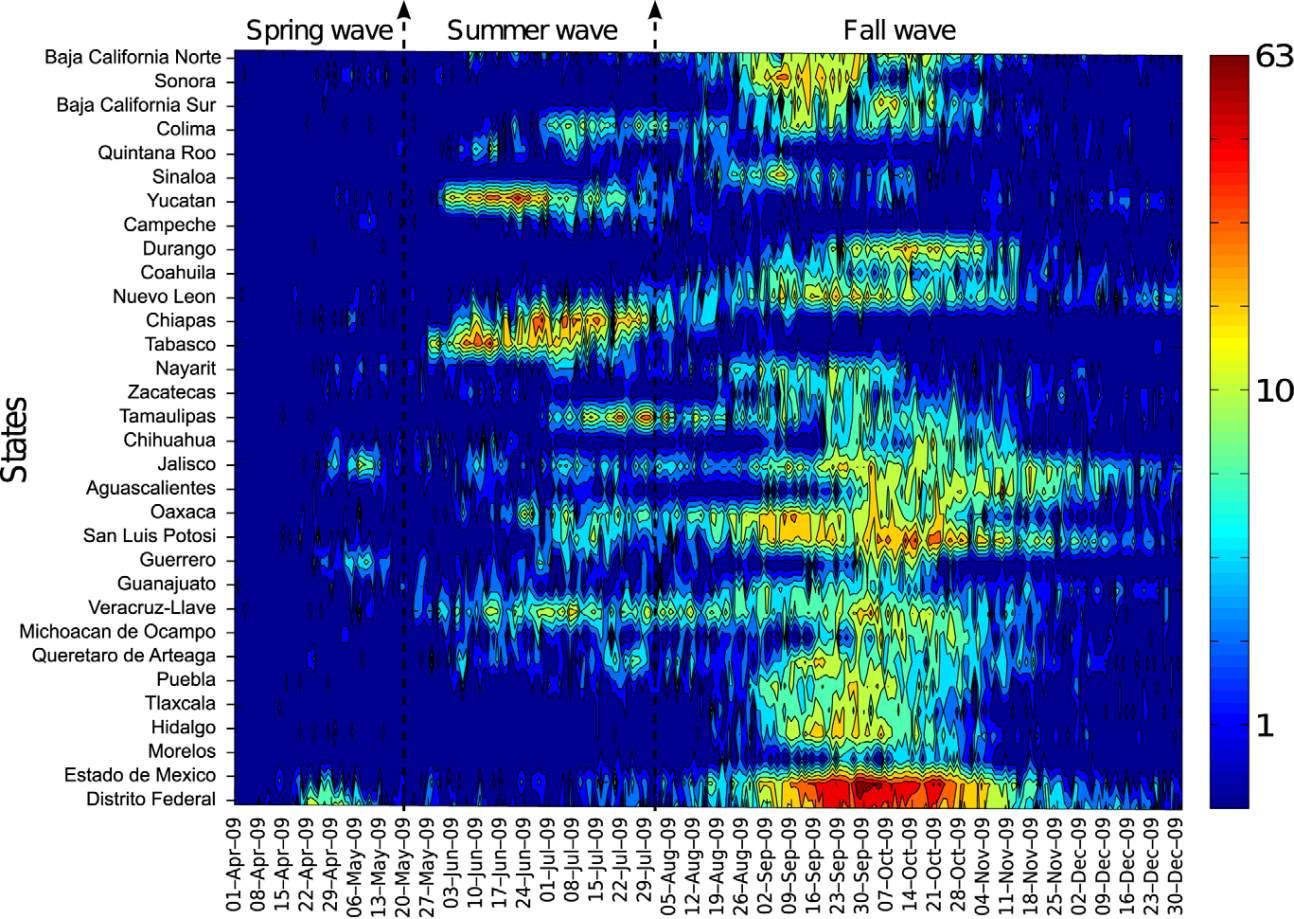
Daily number of laboratory-confirmed A/H1N1 pandemic influenza cases from April 1 to December 31, 2009 in the 32 Mexican states sorted by distance from Mexico City. For visualization purposes, the time series are log-transformed.

We compiled state- and age-specific time series of incident ILI and A/H1N1 pandemic influenza cases by day of symptom onset to analyze the geographic spread of the pandemic across Mexico. We defined three temporally distinct pandemic waves in the spring (April 1–May 20), summer (May 21–August 1), and fall (August 2–December 31) of 2009 on the basis of patterns in national A/H1N1 influenza incidence time series (Figure 1). For each state and pandemic wave, we recorded the cumulative number of cases, cumulative incidence rate, and peak date, defined as the day with the maximum number of new cases.

We also explored geographic variation in the timing of pandemic onset across states and its association with the start of the fall school term, population size, population density, and distance from Mexico City. For each pandemic wave and Mexican state, the onset day was defined as the first day of the period of monotonously increasing cases leading up to the peak of A/H1N1 cases, as in [20].

### Age Distribution of Influenza Cases and Deaths

We examined the age distribution of ILI and A/H1N1 pandemic influenza cases by geographic region and over time, using weekly rather than daily case time series in order to avoid low case counts at the beginning and end of each pandemic wave. We also estimated age-specific measures of disease severity including the case-fatality ratio (CFR  =  deaths/cases, where numerators and denominators can be based on ILI or laboratory-confirmed cases).

### Estimation of Transmission Potential

We estimated the reproduction number, R, for each pandemic wave and geographic region of Mexico (north, central, and southeast). We used a simple method that relies on the estimation of the growth rate by fitting an exponential function to the early ascending phase of daily A/H1N1 pandemic cases, where the epidemic curve is based on symptoms onset (Text S1 and [20]–[23]). The early ascending phase was determined as the period between the day of pandemic onset (as defined above) and the midpoint between the onset and peak days, for each regional pandemic wave. We assumed a mean generation interval of 3 and 4 d, which are within the range of mean estimates for the 2009 A/H1N1 influenza pandemic [11],[13],[24],[25].

We assessed the sensitivity of our estimates to small variations in the definition of the ascending phase used to estimate the exponential growth rate (±4 d). Because variability in daily testing rates could affect R estimates derived from A/H1N1 time series, particularly during the early phase of the spring wave, we conducted a sensitivity analysis using ILI time series.

### Impact of School Closures during the 2009 Spring Wave

School activities have been linked with increased influenza transmission rates in both pandemic and interpandemic periods [26]–[29]. We assessed the effectiveness of mandatory school closures and other social distancing measures implemented during April 24–May 11, 2009 in the central region of Mexico in reducing influenza transmission rates. We fitted a mathematical model of influenza transmission to daily case data (Text S1). This approach allows estimation of separate influenza transmission rates for the periods before and during intervention and explicitly accounts for the depletion of susceptible individuals.

In addition, to analyze changes in the age distribution of cases with school activity periods, we computed the daily ratio of incident A/H1N1 pandemic cases among the student population (5–20 y) to cases among other age groups.

## Results

### General Description of the Three Pandemic Waves in Mexico

A total of 117,626 ILI cases were reported by IMSS from April 1 to December 31, 2009, of which 36,044 were laboratory tested (30.6%) and 27,440 (23.3%) were confirmed with A/H1N1 pandemic influenza. A total of 1,370 ILI deaths (3.6 per 100,000) were reported to the surveillance system, of which 585 (1.5 per 100,000) were confirmed with A/H1N1 pandemic influenza. There was no significant trend in testing rates by geographic region or age group, and testing remained constant over time, except for a rapid increase during the first 2–3 wk of the pandemic (Text S1 and figures B–E therein).

The spatial-temporal distribution of A/H1N1 pandemic influenza and ILI cases reveal a three-wave pattern in the spring, summer, and fall of 2009 with substantial geographical clustering (Figures 1–3). The spring pandemic wave in April–May 2009 was mainly confined to the greater Mexico City area and other central states. The summer wave in June 2009 was limited to southern states, and ended soon after the start of the summer school vacation period on July 3, 2009. A third wave of widespread activity began in August 2009, coinciding with the return of students from summer vacations, and disease activity persisted until December 2009 throughout Mexico.

**Figure 2 pmed-1000436-g002:**
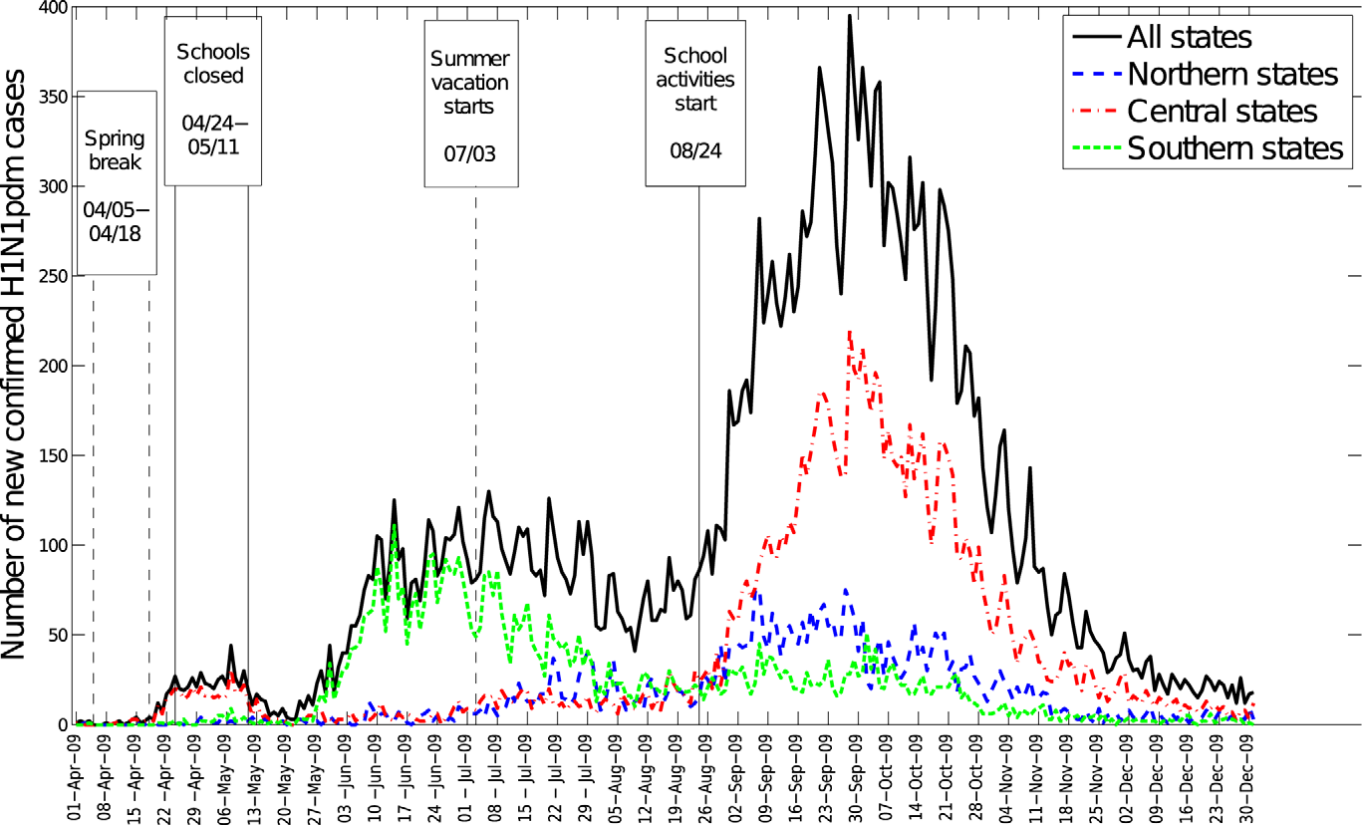
Daily epidemic curve in northern, central, and southeastern states of Mexico, April 1 to December 31, 2009, based on laboratory-confirmed A/H1N1 pandemic influenza cases.

**Figure 3:**
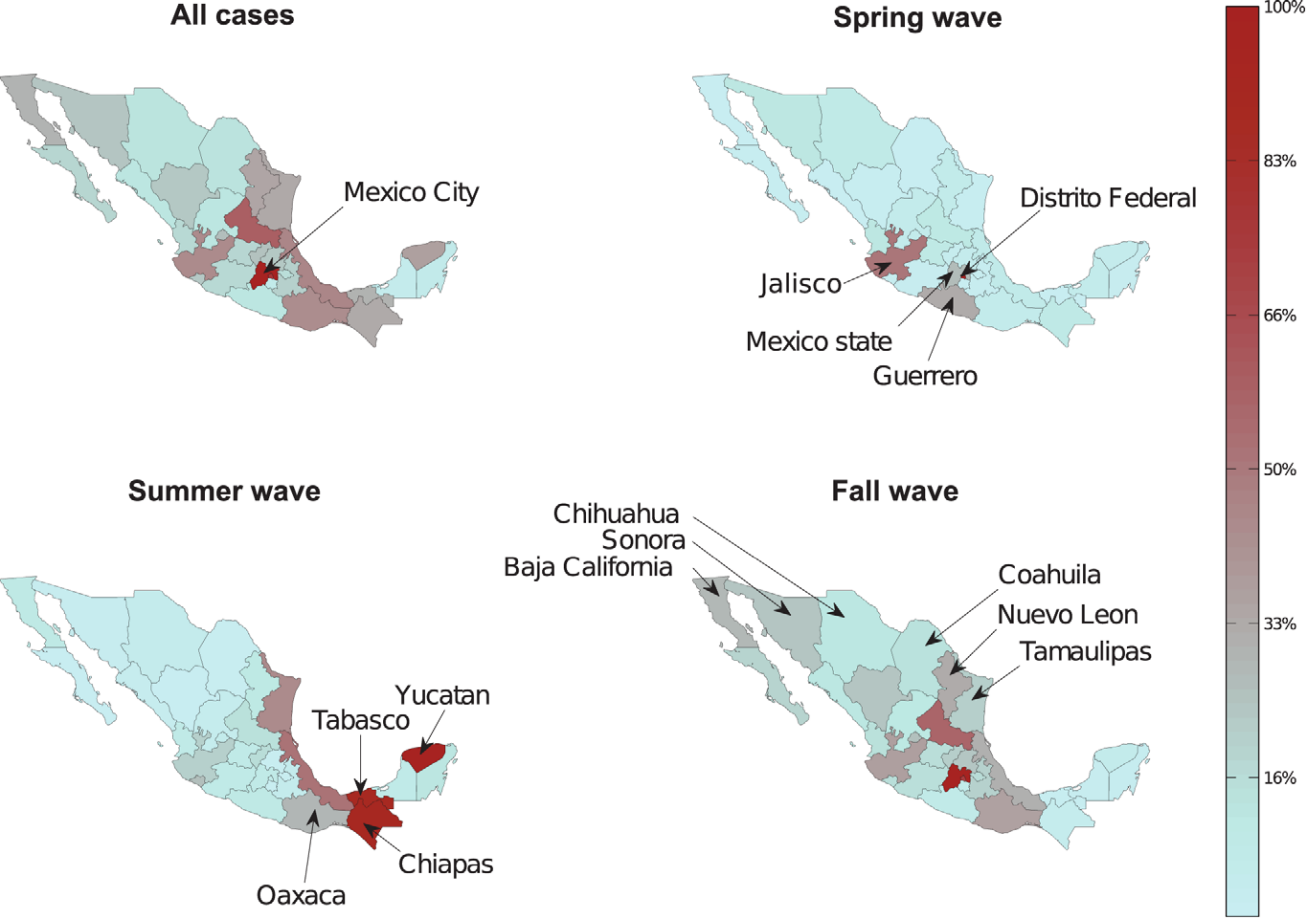
Maps of laboratory-confirmed A/H1N1 pandemic cases across Mexican states for the entire study period, April–December 2009, and by pandemic wave. The spring wave (April 1–May 20) was focused on the central region, including the state of Mexico, Distrito Federal, Jalisco, Puebla, San Luis Potosi, Guerrero, Hidalgo, and Tlaxcala. The summer wave (May 21–August 1) was concentrated in the southeast states of Veracruz, Yucatan, Quintana Roo, Chiapas, Oaxaca, Tabasco, and Campeche. The fall wave (August 2–December 31) affected the central region and the northern states of Baja California Norte, Sonora, Chihuahua, Coahuila, Nuevo Leon, and Tamaulipas. For each pandemic wave, the color scale range was set according to the highest number of cases across states.

The average cumulative incidence rate of pandemic A/H1N1 was 16.6 per 100,000 across the 32 states (95% confidence interval [CI] 16.2–17.0) in spring-summer and 55.7 per 100,000 (95% CI 55.0–56.5) in the fall. Most states experienced highest disease rates in the fall, except for five southeastern states (Figure 3). Similar spatial and temporal patterns were observed in hospitalization and mortality time series (Text S1, figure F).

### Age Patterns of Cases and Disease Severity

The median age of A/H1N1 cases was 18 y (range, 0–99 y). H1N1 morbidity rate was highest among children 5–14 y (115.7 per 100,000) and lowest among seniors 60 y and older (9.2 per 100,000, Table 2; Text S1, figure G). The age-specific risk of severe disease was J-shaped, with highest case-fatality and case-hospitalization rates in people older than 60 y, and relatively high rates in infants (Table 3). The overall CFR was estimated at 1.2% (95% CI 1.1–1.2) on the basis of ILI cases and deaths and 5% (95% CI 4.7–5.3) on the basis of laboratory-confirmed A/H1N1 cases and deaths. The ILI CFR varied geographically and was estimated at 0.5% (95% CI 0.4–0.5) in the southeastern region, 1.0% (95% CI 0.9–1.1) in the northern region, and 1.9% (95% CI 1.8–2.1) in the central region.

**Table 2:** Distribution of age-specific laboratory-confirmed 2009 A/H1N1 pandemic influenza morbidity rates by geographic region in Mexico, April 1–December 31, 2009.

Age (y)	Mexico		Central States		Northern States		Southeastern States	
	Total	Incidence per 100,000	Total	Incidence per 100,000	Total	Incidence per 100,000	Total	Incidence per 100,000
**Total ** ***n***	**27,440**	**72.2**	**10,976**	**71.1**	**4,484**	**44.1**	**6,115**	**126.7**
0–4	3,600	112.7	1,267	106.9	677	72.4	904	235.3
5–14	7,988	115.7	3,254	121.8	1,236	62.8	1,817	226.4
15–29	8,699	115.4	3,356	112.1	1,412	72.2	2,010	192.7
30–44	4,275	48.6	1,804	50.5	684	28.1	857	77.0
45–59	2,340	41.0	1,052	42.8	386	26.7	431	59.1
≥60	538	9.2	243	9.5	89	6.2	96	12.7
Mean ± SD	21.2 ± 16.0	**—**	22.0 ± 16.3	**—**	21.0 ± 16.2	**—**	20.0 ± 15.3	**—**
Median [range]	18 [0–99]	**—**	19 [0–99]	**—**	18 [0–89]	**—**	17 [0–97]	**—**

We note a slight but significant difference in the age distribution of cases between regions (Wilcoxon test, *p<*0.009).

SD, standard deviation.

**Table 3:** Age-specific 2009 A/H1N1 pandemic influenza severity estimates in Mexico, April 1–December 31, 2009.

Age (y)	ILI Cases Hospitalized for Severe Acute Respiratory Infection		Laboratory-Confirmed A/H1N1 Hospitalizations n (A/H1N1 Admission Rate^a^)	ILI Deaths n(Mortality Rate^a^)	Confirmed A/H1N1 Admissions (95% CI)^b^	ILI CFR (95% CI)^c^	Confirmed A/H1N1 CFR (95% CI)^d^	Confirmed A/H1N1 Death Rate among Hospitalized Cases (95% CI)^e^
	*n*	Percent of Total ILI Cases						
**Total**	11,706	10.0 (9.8–10.1)	3,402 (9.0)	1,370 (3.6)	12.4 (12.0–12.8)	1.2 (1.1–1.2)	5.0 (4.7–5.3)	17.2 (15.9–18.5)
0–4	2,399	13.3 (12.8–13.8)	434 (13.6)	109 (3.4)	12.1 (11.0–13.2)	0.6 (0.5–0.7)	3.0 (2.5–3.6)	11.3 (8.3–14.3)
5–14	1,523	5.2 (5.0–5.5)	600 (8.7)	68 (1.0)	7.5 (6.9–8.1)	0.2 (0.2–0.3)	0.9 (0.7–1.1)	5.3 (3.5–7.2)
15–29	2,580	7.4 (7.1–7.7)	992 (13.2)	228 (3.0)	11.4 (10.7–12.1)	0.7 (0.6–0.7)	2.6 (2.3–3.0)	12.6 (10.5–14.7)
30–44	2,277	10.8 (10.4–11.3)	655 (7.4)	383 (4.4)	15.3 (14.2–16.4)	1.8 (1.6–2.0)	9.0 (8.1–9.8)	26.6 (23.1–30.0)
45–59	1,744	16.3 (15.6–17.0)	530 (9.3)	371 (6.5)	22.6 (20.9–24.4)	3.5 (3.1–3.8)	15.8 (14.3–17.3)	28.5 (24.6–32.4)
≥60	1,183	30.6 (29.1–32.1)	191 (3.3)	211 (3.6)	35.5 (31.4–39.6)	5.5 (4.7–6.2)	39.2 (35.0–43.4)	28.3 (21.2–34.8)

a Per 100,000 people affiliated to IMSS.

b (Admitted to hospital with confirmed H1N1/total confirmed H1N1) * 100.

c (Deaths/ILI) *100.

d (H1N1 deaths/ H1N1 cases) *100.

e (H1N1 deaths/H1N1 hospitalizations) *100.

Cumulative rates of A/H1N1 followed a similar age profile across all regions, with peak morbidity rates in the age range of 0–14 y and a consistent drop in morbidity rates after age 30 (Table 2). There was a trend towards increasing age as the fall wave progressed (September 9–December 31; regression against time *R* ^2^ = 0.94, *p<*0.0001), with the median age reaching ∼31 y in December 2009 (Text S1, figure H). There was a similar trend in ILI cases (*R* ^2^ = 0.94, *p<*0.0001), laboratory-confirmed hospitalized cases (R^2^ = 0.62, *p = *0.0002), and laboratory-confirmed deaths (*R* ^2^ = 0.26, *p = *0.04).

### Demographic Factors and Variation in Timing and Magnitude of the Pandemic

Next we explored whether demographic factors may partly explain the observed variation in timing of onset and magnitude of the three pandemic pandemic waves across the 32 Mexican states. First, we tested the association between the incidences of successive waves, which could reflect the gradual build-up of immunity (and thus, negative association) or the impact of baseline sociodemographic factors (positive association). Cumulative incidence rates had a weak positive correlation between spring and fall (Spearman rho for A/H1N1 rates  =  0.4, *p = *0.046), but there was no significant correlation between the summer wave and the spring or fall waves (*p*>0.16).

The total morbidity burden of the pandemic, measured as the cumulative A/H1N1 incidence rate during April–December 2009, was negatively correlated with population size (Spearman rho = −0.58, *p<*0.001, Text S1 and figure I therein). We found a similar correlation with ILI rates and rates of IMSS-affiliated individuals tested for influenza (Spearman rho = −0.4, *p = *0.02, and rho = −0.61, *p<*0.001, respectively) and the association remained after adjustment for population structure. These findings suggest that low population areas reported higher pandemic morbidity rates than large population centers and that the association was not an artifact of testing practices or population age structure. In contrast, we did not find any association between pandemic morbidity rates and population density. Further, rates of hospitalization and death were not correlated with population size or density (*p*>0.15).

Population size was also associated with the onset of the fall pandemic wave, with earlier onset occurring in more populous states (Spearman rho = −0.60, *p = *0.003; Text S1, figure J); however, there was no association between onset and population density (rho = −0.032, *p = *0.13), distance from Mexico City (rho = 0.02, *p = *0.92), or the onset of earlier waves (Text S1).

### Trends in Reproduction Number (R) and Impact of School Closure

We estimated the mean R for the spring, summer, and fall waves in three geographic regions based on confirmed H1N1 cases (Table 4; Text S1, figure K). Assuming a mean generation interval of 3 (and 4) d, the mean R was estimated to be 1.8 (2.1) for the spring wave in the central region prior to the national school closure period, 1.6 (1.9) for the summer wave in the southeast region, and 1.2 (1.3) for the fall wave in both central and northern regions. R estimates obtained from ILI cases were 13%–17% lower than those obtained from confirmed cases for the spring and summer waves, while there was no difference for the fall wave. There was little variation in R estimates when we increased or shortened the growth rate period by 4 d (difference of 0.1–0.2 for the spring and summer waves and 0.1 or less for the fall wave). An upper bound for R is provided in Text S1, table B, with the extreme case of a fixed generation interval, and suggests that R remained below 2.5 throughout the pandemic in Mexico.

**Table 4:** Mean estimates of the reproduction number and corresponding 95% CIs for the spring, summer, and fall waves of the 2009 A/H1N1 influenza pandemic by geographic region.

Pandemic Wave	Geographic Region					
	Central States		Southeastern States		Northern States	
	3-d Serial Interval	4-d Serial Interval	3-d Serial Interval	4-d Serial Interval	3-d Serial Interval	4-d Serial Interval
**Spring**	1.80 (1.78–1.81)	2.12 (2.09–2.14)	—	—	—	—
**Summer**	—	—	1.62 (1.61–1.63)	1.85 (1.84–1.87)	—	—
**Fall**	1.23 (1.22–1.23)	1.31 (1.30–1.31)	—	—	1.24 (1.23–1.24)	1.32 (1.31–1.32)

The latent and infectious periods are assumed to be exponentially distributed with a serial interval of 3 and 4 d. The epidemic growth phase used to estimate the R consisted of 14 d for the spring wave (April 12–April 25) and summer wave (May 21–June 3) and 28 d for the fall wave in central states (August 5–September 1) and northern states (August 8–September 4).

See Text S1, figure K, for exact time periods considered as part of the epidemic growth phase.

**Figure 4:**
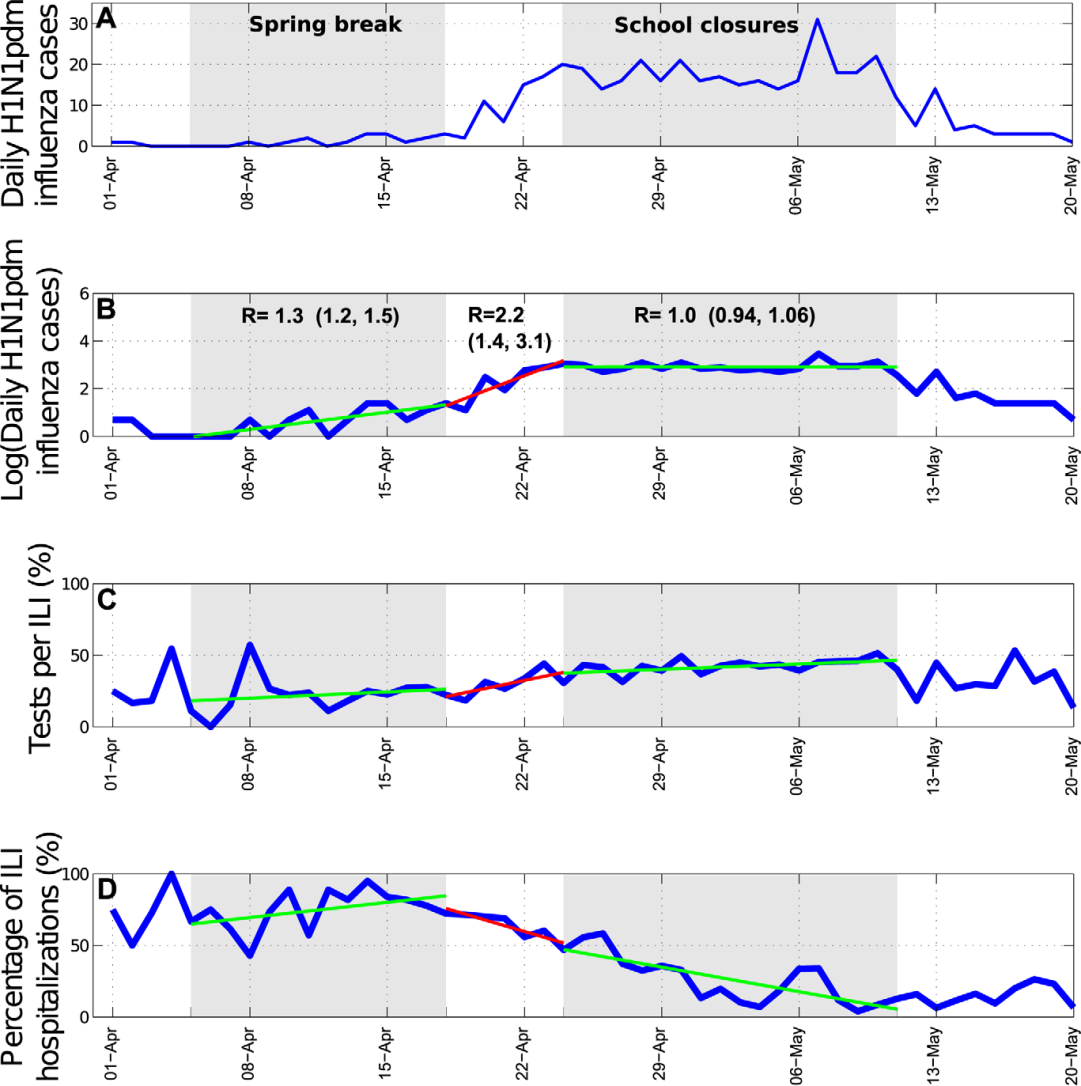
Trends in influenza pandemic patterns and school activities. (A) H1N1 cases, natural scale; (B) H1N1 cases, log-scale, (C) testing rates (*n* tests/*n* ILI), and (D) proportion of hospitalizations among ILI cases during the spring pandemic wave in central Mexico in 2009. Shaded areas denote periods when schools are not in session, including during the spring break (April 4–18) and the mandatory suspension of educational activities (April 24–May 11). (B) indicates changes in the R estimates over time, as measured from the exponential growth rate of the incidence curves.

**Figure 5:**
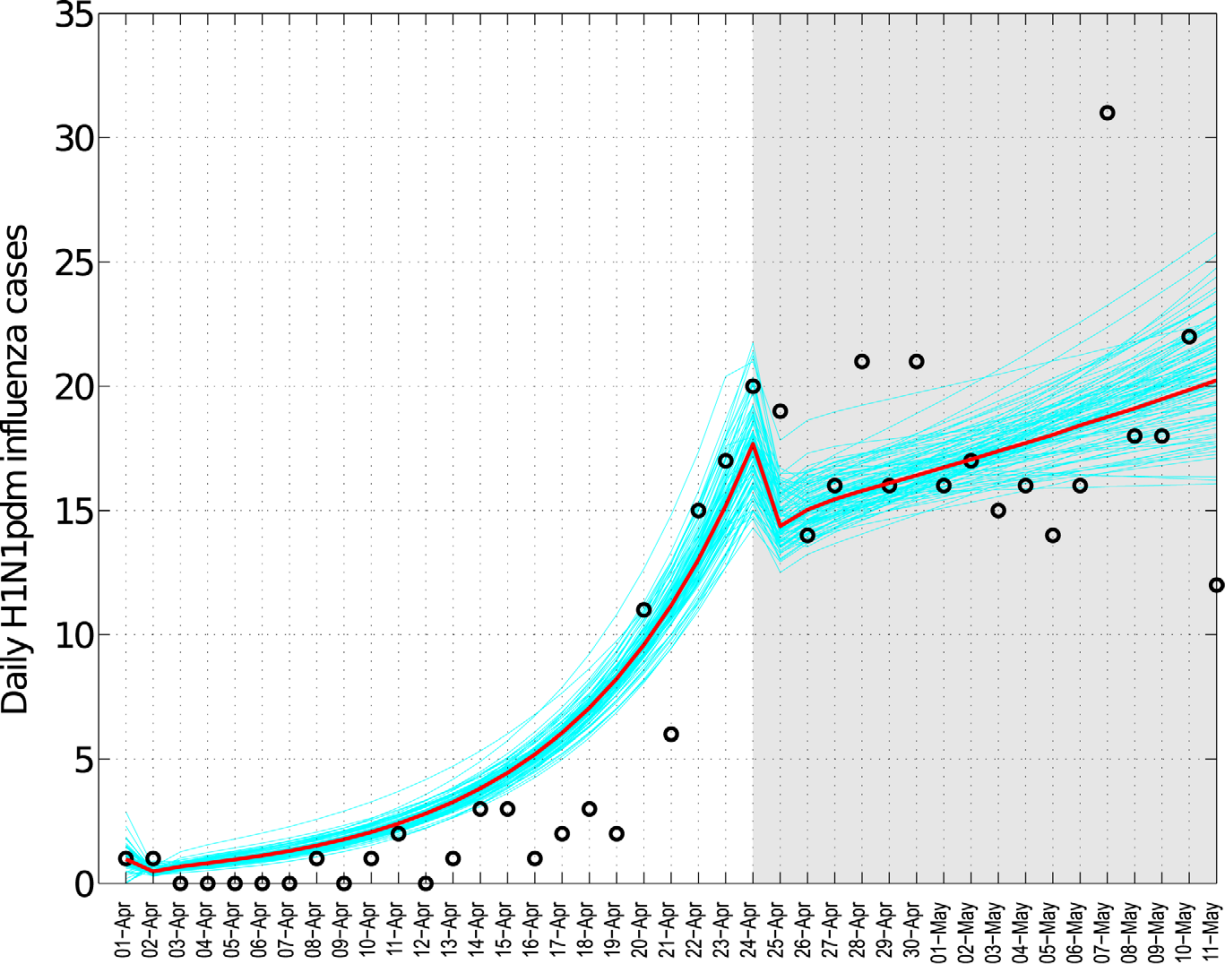
Fit of influenza transmission model to the daily number of H1N1 pandemic influenza cases in central Mexico, April 1–May 11, 2009. The grey shaded area indicates the suspension of educational activities and other social distancing measures implemented between April 24 and May 11, 2009. Black circles represent the observed data. The solid red line is the model best-fit, and the blue lines are CIs based on 100 realizations of the model obtained by parametric bootstrapping (Text S1).

We identified significant changes in the R during the spring wave according to school activity periods (Figure 4A and 4B). Focusing on central states affected by a substantial spring wave, we estimate that R increased from 1.3 (95% CI 1.2–1.5) to 2.2 (95% CI 1.4, 3.1) after the end of the spring break vacation period. A decrease in R from 2.2 (95% CI 1.4–3.1) to 1.0 (95% CI 0.94–1.06) coincided with the suspension of educational activities and the implementation of other social distancing measures enforced between April 24 and May 11, 2009. To explicitly account for the effects of depletion of susceptible individuals, we fitted a transmission model to daily influenza H1N1 case data and quantified the relative change in mean transmission rate during the intervention period. We estimated that the transmission rate was reduced by 29.6% (95% CI 28.9%–30.2%) during the intervention period (Figure 5). Our model gave a good fit to the spring epidemic curve overall, although it yielded a slightly higher number of cases than observed until the last week of April (chi-square test, bins  =  41, df = 37, *p = *0.22, Figure 5). As a sensitivity analysis, we also fitted the model to ILI cases and found a reduction of 36.2% (95% CI 35.9%–36.5%) associated with social distancing measures.

**Figure 6:**
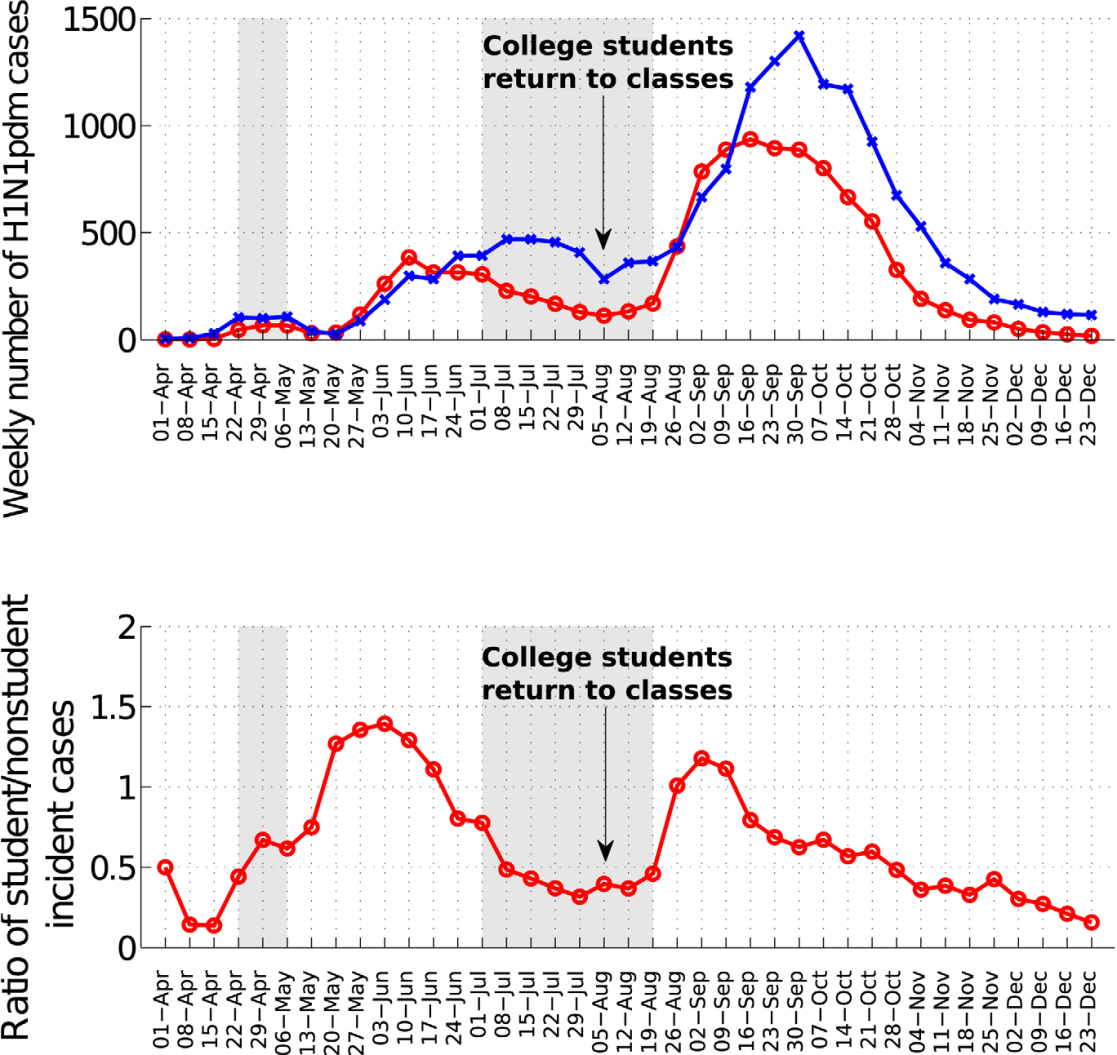
Changes in the age distribution of cases during the summer and fall pandemic waves in Mexico. (A) Weekly time series of laboratory-confirmed A/H1N1 pandemic cases among students (5**–**20 y, red curve) and other age groups (blue curve) and (B) Weekly ratio of student to nonstudent A/H1N1 cases. The grey shaded area indicates the mandatory school closure period (April 24**–**May 11) and the summer vacation period (July 3**–**August 23) for elementary and secondary school students. College students retuned to class on August 10th(arrow).

To further test the impact of school cycles, we monitored trends in the ratio of incident student to nonstudent influenza A/H1N1 cases. At the national scale, this ratio was low during the summer vacations and increased sharply following the start of school activities in August (Wilcoxon test, *p<*0.001, Figure 6). At the state level, the ratio of student to nonstudent cases peaked 2**–**5 wk after schools reopened in the fall of 2009 (Text S1, figures L**–**M).

## Discussion

This is, to our knowledge, the first study to explore spatio-temporal variation in the dynamics and age patterns of the 2009 A/H1N1 pandemic in Mexico, relying on a large sample of laboratory-confirmed and ILI data collected by a private medical system representing a population of over 100 million people. Our findings support the effectiveness of early mitigation efforts in the greater Mexico City area in the spring of 2009, including mandatory school closures and cancellation of large public gatherings. In addition, the onset of the fall pandemic wave in Mexico coincided with the start of the fall term in schools and universities, reinforcing the importance of school cycles in the transmission of pandemic influenza. Our data also reveal substantial geographical variation in pandemic patterns across Mexico, in part related to population size, with three consecutive waves of varying amplitude occurring over an 8-mo period. In line with previous studies [**30**]–[**32**], we note that the age distribution of pandemic influenza morbidity was highly skewed towards younger age groups (median 18 y), while the risk of severe disease was skewed towards older age groups. Of note was the particularly high CFR reported in these Mexican data (CFR≈1% based on the ratio of ILI deaths to ILI cases).

Our transmission model fitted to daily case data suggests that the 18-d period of mandatory school closure and cancellation of public gathering in the greater Mexico area was associated with a 29%**–**37% reduction in the transmission of pandemic influenza. Overall, our estimates are in agreement with a recent study suggesting an ∼25% reduction in A/H1N1 transmission following secondary schools closures in Hong Kong from June 11 to July 10, 2009 [33]. Similarly, a European study of variation in contact rate patterns suggested a 13%**–**40% reduction in reproduction number with holiday periods in Belgium, Great Britain, and The Netherlands [34]. In our data, the resurgence of influenza activity within 2**–**5 wk of the beginning of fall term in the 32 Mexican states, together with a rapid change in the age distribution of cases around this time, further suggests the importance of school cycles for pandemic influenza transmission. Accordingly, previous studies have shown a temporal association between school cycles and the onsets of the fall 1957 and 2009 pandemic waves in the US [28],[35],[36].

While past studies have concentrated on R estimates for the spring wave in Mexico and other countries, this is the first study, to our knowledge, to provide estimates for all three pandemic waves in any country. Our estimates were highest for the spring wave (R≈1.8**–**2.1), declined in the summer (R≈1.6**–**1.9), and were lowest in the fall (R≈1.2**–**1.3). The significantly lower fall estimates may be explained by higher levels of herd immunity and preventive measures put in place in preparation for the start of school term. These included cleaning and disinfection of schools, promotion of hand hygiene, and screening and management of incident ILI cases among students and school staff.

Our R estimates were robust to small variation in the definition of the epidemic ascending phase and the use of confirmed H1N1 or ILI cases, which rules out potential biases due to testing practices. Overall our spring R estimates are in line with previous studies focusing on the early pandemic phase in Mexico, with estimates ranging between 1.4**–**2.4 [11],[37],[38]. In other countries, R was estimated at 1.2**–**2.4 for community-based settings in Japan [39], New Zealand [40], Australia [41], Peru [42], Chile [43], Ontario, Canada [44], and the US [45], while higher estimates in the range 2.3–3.3 were obtained during school outbreaks [13],[14],[46]. The variability in published R estimates of the first wave of the 2009 pandemic could be attributed to differences in control strategies, school activity periods, travel patterns, and climatic conditions [8],[9], which should be more fully investigated.

The number and intensity of the 2009 H1N1 pandemic waves varied substantially across regions of the world. While Mexico, the US, and the UK experienced a “herald” pandemic wave in the spring of 2009 followed by one or more waves during the summer and fall 2009 [10],[12],[47], a number of countries, particularly in the Southern Hemisphere, have experienced only a single pandemic wave in 2009, including Chile [48], Argentina [49], Australia [50],[51], and New Zealand [50]. Other countries in Europe also experienced a single main wave in the fall of 2009 [16], followed by a recrudescence of H1N1 activity more than one year later in winter 2010–2011. Our detailed analysis of 2009 pandemic patterns in Mexican states suggests that all of these configurations were observed within Mexico. Similarly, past influenza pandemics have exhibited multiple waves over short periods of time, as reported for the 1918 pandemic in Mexico [22] and elsewhere [52]–[54].

For reasons that remain unclear, there are substantial spatial variations in the seasonality of influenza epidemics across Mexican regions in interpandemic years, which may have played a role in the geographical asynchrony of the 2009 A/H1N1 pandemic. Interpandemic influenza activity has strong winter seasonality in northern and central Mexico [1], while influenza has been detected between December and July in the tropical southeast [55]. It is perhaps not surprising that the Southeast region experienced a large-scale A/H1N1 pandemic wave in summer 2009 and a relatively minor wave in the fall. While absolute humidity has been found to be associated with the onset of interpandemic and pandemic influenza activity in the US [9],[56], we did not identify a correlation with the three-wave pandemic profile in Mexico (Text S1) [56]. Further analysis of the environmental or social factors influencing the transmission of interpandemic and pandemic influenza is warranted in order to fully explain influenza seasonality patterns [57].

We found that spatial variation in the timing and magnitude of the three A/H1N1 pandemic waves across Mexican states was partly linked to population size. Influenza spread in Mexico was driven by large population centers, reminiscent of seasonal influenza in the US [58] and the 1918 pandemic in England and Wales [20],[59]. We found significant spatial heterogeneity in the distribution of incidence rates across states, with lowest incidence rates observed in large population centers. A similar protective effect of large population centers was evidenced in the context of the 1918 pandemic in England and Wales [20]. These results could be explained by local differences in health care seeking behavior or in the effectiveness of social distancing measures [60].

Our large dataset allowed estimation of pandemic disease severity for relatively fine age groups, which could help identify priority age groups for vaccination and treatment in future pandemics. Although it may not be possible to extrapolate findings from this pandemic to the next influenza pandemic, the last four pandemics have been characterized by significant excess mortality among young adults as well as significant sparing of older populations [52]. Our case-based severity estimates derived from hospitalization and death reports were highest among people older than 60 y, and they were substantially higher than in other countries [32],[61]–[64]. In particular, our CFR based on ILI visits was estimated at 3% during the spring wave, 0.5% during the summer wave, and 1.2% during the fall wave, while our ILI-based hospitalization rate was around 10%. This is one to two orders of magnitude higher than estimates reported in several studies [61],[62],[64] and similar to estimates based on hospitalization cases series in the spring of 2009 in California and Argentina [63],[65]. Our high case-based severity estimates likely reflect a bias of the Mexican IMSS influenza surveillance system towards the higher levels of the severity pyramid [62]. As a sensitivity analysis, and for comparison with previous studies, we estimated CFR using 2009 A/H1N1 serological attack rates as denominator. Because of the lack of serological estimates from Mexico, we used age-specific serological data from the UK reported for the two waves of the pandemic there (May 2009 to April 2010) [66]. Using UK data as denominator suggests that the age-adjusted CFR could be in the order of ∼0.01% in Mexico with a pattern of increasing severity with age. This estimate is two orders of magnitude lower than our CFR based on ILI cases and is in close agreement with estimates from other countries [61],[62],[64]. Further studies comparing excess mortality rates derived from vital statistics for different countries and influenza seasons may shed more light on the relative severity of this pandemic.

Several caveats are worth noting in our analysis of the 2009 pandemic in Mexico. We used data on ILI and laboratory-confirmed influenza cases reported to the Mexican Institute for Social Security network in 32 states, and there may be sampling variation between states. However, about one-third of all ILI cases were consistently tested for influenza in all regions and throughout the main pandemic period (except for the early spring), and we did not see any evidence of weaker disease surveillance in smaller states (Text S1). On the contrary, states with lower population sizes reported more cases proportionally than larger states. The reduction in R observed during the social distancing period occurred during a period of increasing testing rates (Figure 4C). One would expect that increasing testing rates would lead to overestimation of the growth rate in H1N1 cases and may in turn result in overestimation of the impact of social distancing. Nevertheless, our sensitivity analyses based on ILI data gave similar results, and we do not think likely that spatial or temporal differences in ILI rates and health-seeking behavior may bias these analyses. We cannot rule out, however, the impact of other factors on R estimates, including a reduction in the delay from symptom onset to hospital admission in the spring, potentially reducing the effective infectious period (Figure 4D) [17], and the use of 1.2 million doses of oseltamivir for influenza treatment around the time of school closure.

In conclusion, our work suggests that intervention measures initiated in Mexico early in the pandemic period in April–May 2009 were effective in temporarily reducing disease transmission and that the start of the fall school term in August 2009 may have facilitated the onset of a widespread pandemic wave. It will be interesting to formally compare the Mexican experience with that of other locations that applied similar measures, such as Hong Kong [33]. The heterogeneous Mexican experience also suggests that it will be relatively difficult to predict the local impact and transmission dynamics of future influenza pandemics globally. We suggest that population size and school cycles can account for some of the observed variability and should be integrated into future pandemic planning scenarios. Finally, it is important to keep in mind that several post-1918 pandemic waves were associated with substantial health impact in the Americas [22],[67] and that the majority of influenza deaths associated with the 1889 pandemic in London occurred 2 y after the initial wave [68]. Therefore, we must remain vigilant and continue to monitor the circulation and health burden of the A/H1N1 pandemic virus in the coming years [69].

## Supporting Information

Alternative Language Abstract S1 Spanish translation of the Abstract by GC.
(0.01 MB DOCX)

Text S1 Characterizing the epidemiology of the 2009 influenza A/H1N1pandemic in Mexico: Supplementary information.
(0.37 MB PDF)

## Abbreviations

CFR: case-fatality ratio
CI: confidence interval
ILI: influenza-like illness
IMSS: Mexican Institute for Social Security
R: reproduction number
