# Supplementary material for: Characterizing the Epidemiology of the 2009 Influenza A/H1N1 Pandemic in Mexico
Source: PLoS Med. 2011 May 24;8(5):e1000436. doi: 10.1371/journal.pmed.1000436 (PMC3101203; doi:10.1371/journal.pmed.1000436)
Supplement: Alternative Language Abstract S1 — Spanish translation of the Abstract by GC. (0.01 MB DOCX) [file pmed.1000436.s001.docx]

***Antecedentes:*** Las autoridades locales y nacionales de México iniciaron una intensa respuesta de salud pública durante las primeras etapas de la pandemia de influenza A/H1N1 en 2009. Aquí se analizan los patrones epidemiológicos de la pandemia en abril-diciembre de 2009 en México y se evalúa el impacto de las intervenciones no farmacéuticas, los ciclos escolares y los factores demográficos en la transmisión de la pandemia.

***Métodos:*** Se utilizaron los datos de vigilancia de la influenza recolectados por el Instituto Mexicano del Seguro Social, que representa el 40% de la población, para estudiar los patrones de enfermedad tipo influenza (ILI, por sus siglas en inglés), hospitalizaciones, muertes, y la tasa de letalidad para cada ola pandémica y región geográfica. También se estimó el número reproductivo (R) basado ​​en la tasa de crecimiento de la serie de casos diarios, y se utilizó un modelo de transmisión para evaluar la eficacia de las estrategias de mitigación iniciadas durante la primera ola de la pandemia.

***Resultados:*** Un total de 117,626 casos ILI fueron identificados entre abril y diciembre de 2009, de los cuales el 30.6% se les realizaron pruebas para la influenza, y el 23.3% fueron positivos al nuevo virus H1N1. Un perfil de pandemia de tres olas se identificó, con una ola inicial en abril-mayo (zona metropolitana de la Ciudad de México), una segunda ola en junio-julio (estados del sureste), y una tercera ola geográficamente extendida en agosto y diciembre. La edad media de casos ILI confirmados por laboratorio fue de ~ 18 años y se incrementó a ~ 31 años durante el otoño (p <0,0001). La tasa de letalidad entre los casos de ILI fue de 1.2%, y la más alta (5.5%) ocurrió entre las personas mayores de 60 años. Las estimaciones regionales del número reproductivo fueron de 1.8-2.1, 1.6-1.9 y 1.2-1.3 para las olas de primavera, verano y otoño, respectivamente. Estimamos que el cierre mandatorio de las escuelas durante 18 días y otras medidas de distanciamiento social aplicadas en el área metropolitana de la Ciudad de México se asoció con una reducción del 29-37% en la transmisión de la influenza en la primavera de 2009. Además, un aumento en el número reproductivo se observó a finales de mayo y principios de junio en los estados del sureste, después de que la suspensión escolar obligatoria se reanudara y antes del inicio de las vacaciones de verano. La segunda ola pandémica en los estados comenzó 2-5 semanas después de empezar las actividades escolares para el semestre de otoño que coincidió con un cambio en la edad de los casos de influenza.

***Conclusiones:*** Hemos documentado tres olas espacialmente heterogéneas de la pandemia del virus H1N1 de 2009 en México, que se caracterizaron por una distribución de edad joven de los casos. Nuestro estudio realza la importancia de los ciclos escolares en la dinámica de transmisión de esta pandemia de influenza, y sugiere que el cierre de escuelas y otras medidas de mitigación pueden ser útiles para mitigar futuras pandemias de influenza.
