# Supplementary material for: Characterizing the Epidemiology of the 2009 Influenza A/H1N1 Pandemic in Mexico
Source: PLoS Med. 2011 May 24;8(5):e1000436. doi: 10.1371/journal.pmed.1000436 (PMC3101203; doi:10.1371/journal.pmed.1000436)

Characterizing the epidemiology of the 2009 influenza A/H1N1pandemic in Mexico:

Supplementary Information

G. Chowell, S. Echevarría-Zuno, C. Viboud, L. Simonsen, J. Tamerius, M. A. Miller, V.H. Borja Aburto

1. Methods:

1.1 Estimation of the reproduction number

1.2 Influenza Transmission Model to evaluate the effectiveness of intervention strategies in the Spring of 2009 in the Central region of Mexico

2. Results

2.1 Trends in testing and influenza-positivity rates.

2.2 Pandemic morbidity rates, timing of onset, and demographic factors

2.3 Timing of pandemic onset and absolute humidity

References

Supplementary Tables A and B

Supplementary Figures A-N

1. METHODS

1.1 Estimation of the reproduction number

To estimate the reproduction number, R, we first estimated the growth rate "r" by fitting an exponential function to the early ascending phase of daily H1N1pdm cases, where the epidemic curve is based on symptoms onset. The early ascending phase was determined as the period

between the day of pandemic onset and the midpoint between the onset and peak days, for each regional pandemic wave. The reproduction number was derived by substituting the estimate for "r" into an expression derived from the linearization of the classical Susceptible-Exposed-Infectious-Recovered (SEIR) transmission model [1,2]:

$$R=(1+\frac{r}{b_1})(1+\frac{r}{b_2}) \qquad (1)$$

where 1/b1 and 1/b2 are respectively the mean latent and infectious periods which are assumed to be exponentially distributed. Hence, the mean generation interval between two successive cases is given by $T_c = 1/b_1 + 1/b_2$. We assumed a mean generation interval of three ($1/b_1$ =1.5 days and $1/b_2$=1.5 days) and four days ($1/b_1$ =2 days and $1/b_2$=2 days), which are within the range of mean estimates for the 2009 influenza pandemic.

As a sensitivity analysis, we also obtained an upper bound estimate for the extreme case of a fixed generation interval (delta distribution), using the following expression [1]:

$$R = e^{rT_c} \qquad (2)$$

1.2. Influenza Transmission Model to evaluate the effectiveness of intervention strategies in the Spring of 2009 in Central states

We used a simple SEIR (susceptible-exposed-infectious-recovered) transmission model that considers different transmission rates prior to and during the period of social distancing measures implemented in the greater Mexico City area from April 24 to May 10, 2009. The model derives from a mass-action SEIR model previously developed to estimate the transmissibility of seasonal influenza [3,4]. In the "SEIR" model, the population is divided in five categories: Susceptible (S),

Exposed (E), Infectious (I), and removed (P). In this model, infection is transmitted between infectious and susceptible individuals, and no particular route of transmission is assumed [3]. The total population size (N) is assumed constant during the entire Spring pandemic wave in Mexico and fixed according to the current population size estimate for central Mexico (N= 15,439,165). We assume homogeneous mixing, that is, each individual has the same probability of having contact with any other individual in the population. Susceptible individuals infected with the virus enter the latent period (category E) at the rate $\beta(t)$ I/N where $\beta(t)$ is the mean transmission rate per day and at time t. Note that $\beta(t)$ represents a generic transmission rate that combines the effect of direct and indirect contacts. We assumed that the transmission rate remained constant prior to the start of the school closure period (modeled by parameter β_1), and changed to β_2 during the intervention period (where $\beta_2 < \beta_1$ if interventions were effective). The fraction I/N is the probability of contacting an infected individual out of the total population size N. Latent individuals progress to the infectious class at the rate κ ($1/\kappa$ is the mean latent period). The mean infectious period is given by $1/\gamma$. Recovered individuals are assumed protected for the duration of the spring wave. The system of differential equations that describes the above transmission process is given by:

$$dS/dt = -\beta(t)\, S\, I/N$$

$$dE/dt = \beta(t)\, S\, I/N - \kappa E$$

$$dI/dt = \kappa E - \gamma I$$

$$dP/dt = \gamma I$$

and the transmission rate is given by the following function:

$$\beta(t) = \begin{cases} \beta_1 & \text{t} < \text{t}_{\text{school closure}} \\ \beta_2 & \text{t} >= \text{t}_{\text{school closure}} \end{cases}$$

where $t_{school\ closure}$ is the day of the start of the school closures.

The system of ordinary differential equations was solved numerically using Matlab (The Mathworks, Inc).

The reproduction number

The average number of secondary cases generated by a primary infectious case during its period of infectiousness in an entirely susceptible population is known as the basic reproduction number R_0 [5,6]. From our model, the basic reproduction number R_0 is given by the product of the initial transmission rate β_1 and the mean infectious period $1/\gamma$; that is, $R_0 = \beta_1/\gamma$.

Parameter estimation

The mean latent period was fixed to 1.5 days and the infectious period was estimated from the data (bounded to the range 1.5-4 days). To estimate the value of unknown parameters, we rely on the general approach of "trajectory matching", where one searches for the combination of model parameters that produces an epidemic curve most statistically similar to the observed one [7,8]. Based on a study of the 2009 H1N1pdm influenza pandemic in Hong Kong [9], we assumed that the daily number of confirmed H1N1pdm influenza cases represents only 5% of the total incidence of H1N1pdm influenza during the Spring wave.

The mean transmission rates before and during the period of social distancing measures (β_1 and β_2) and the initial numbers of individuals in the exposed E(0) and infectious I(0) categories were estimated by least squares fitting of the model to the daily number of new H1N1pdm influenza cases. Due to the short latent period for influenza (1-2 days) [10,11], we assumed E(0) = I(0); this simplification allowed us to estimate only four parameters from the time series of H1N1pdm cases. The reproduction number (R) was estimated using data comprising the pandemic period preceding the start of the school closure period on April 24, 2009. The mean infectious period was

estimated at 1.5 days.

Uncertainty analyses

We estimated the uncertainty of the estimated model parameters via parametric bootstrap [3,7]. Briefly, we simulated 100 alternate realizations of the epidemic trajectory, by perturbation of the best-fit curve of daily number of new H1N1pdm influenza cases. We added to the best-fit curve a simulated error structure computed using the increment in the “true” number of cases from day j to day j+1 as the Poisson mean for the number of new cases observed in the j to j+1 interval. The 95% bootstrap-based confidence intervals for the reproduction number should be interpreted as containing 95% of estimates if the analysis was repeated with the same model assumptions and if observational error was the only source of noise.

2. RESULTS

2.1 Trends in testing and influenza-positivity rates.

A total of 109,043 ILI cases were reported by IMSS from April 1 to December 31, 2009. Of these, 36,044 were laboratory tested (33.1%), and 27,440 (25.2%) were confirmed with H1N1pdm influenza. The average PCR positivity was 67.4% (95% CI: 61, 73.7; see also Figure D for time trends in PCR positivity rate). Daily incidence curves of ILI and H1N1pdm influenza cases by geographic region had very similar temporal patterns (Spearman rho=0.91-0.98, P<0.0001; Figure E).

Overall testing rates remained stable over the entire pandemic period with an average testing rate of 31% (95% CI: 29, 33). Weekly variation in testing rates across pandemic waves and geographic regions did not reveal significant trends except for the initial increase in testing rates during the very early pandemic phase in the spring (Figure B). Similarly, testing rates were consistent across age groups (range: 30-33%, chi-square test; P=0.99, Figure C).

2.2 Pandemic morbidity rates, timing of onset and demographic factors

Given the highly skewed age patterns of this pandemic, we hypothesized that the proportion of children in each state could partially explain the association between cumulative pandemic mortality rates and population size (Spearman rho = -0.58, P<0.001). The association between H1N1pdm morbidity rates and population size remained statistically significant even after adjusting for the proportion of children (5-14 y) in each state (Spearman partial rho= -0.57, P<0.001), which suggests that population size was truly associated with pandemic morbidity rates in Mexico.

By contrast to the fall wave, the timing of onset of the spring and summer waves was not associated with population size, population density or distance from Mexico City. The timing of the pandemic peak was not associated with any of these factors in any of the 3 waves.

2.3 Timing of pandemic onset and absolute humidity

Absolute humidity was recently linked to influenza activity in the US, whereby low levels of absolute humidity favors influenza transmission and virus survival, and may facilitate the onset of influenza epidemics and pandemics [14-16]. We explored the relationship between the daily variation in absolute humidity and the temporal profile of the 2009 pandemic in the 32 Mexican states. For this purpose, we analyzed daily variation in number of new H1N1pdm influenza cases and the average specific humidity in Mexico weighted by population size, April 1 to December 31, 2009. Daily specific humidity (g/kg), a measure of absolute humidity, was obtained for each state population center from daily averages of temperature, relative humidity and surface pressure obtained from the National Center for Environmental Prediction-National Center for Atmospheric Research (NCEP-NCAR) global reanalysis [12]. We did not find any asosciation between timing of onset of the Spring 2009 pandemic wave at the state level and changes in absolute humidity 1-30 days before pandemic onset (Spearman rho=-0.07 – 0.46, P>0.29, Figure N). Similarly, the onset of the fall pandemic wave occurred during the period of highest absolute humidity levels for the entire year 2009 (Figure N).

References

1. Wallinga J, Lipsitch M (2007) How generation intervals shape the relationship between growth rates and reproductive numbers. Proc Biol Sci 274: 599-604.
2. Lipsitch M, Cohen T, Cooper B, Robins JM, Ma S, et al. (2003) Transmission dynamics and control of severe acute respiratory syndrome. Science 300: 1966-1970.

3. Chowell G, Miller MA, Viboud C (2008) Seasonal influenza in the United States, France, and Australia: transmission and prospects for control. Epidemiol Infect 136: 852-864.
4. Chowell G, Viboud C, Simonsen L, Miller M, Alonso WJ (2010) The reproduction number of seasonal influenza epidemics in Brazil, 1996-2006. Proc Biol Sci 277: 1857-1866.
5. Anderson RM, May RM (1991) Infectious diseases of humans. Oxford: Oxford University Press.
6. Diekmann O, Heesterbeek J (2000) Mathematical epidemiology of infectious diseases: model building, analysis and interpretation: Wiley.
7. Chowell G, Ammon CE, Hengartner NW, Hyman JM (2007) Estimating the reproduction number from the initial phase of the Spanish flu pandemic waves in Geneva, Switzerland. Math Biosci Eng 4: 457-470.
8. Chowell G, Nishiura H, Bettencourt LM (2007) Comparative estimation of the reproduction number for pandemic influenza from daily case notification data. J R Soc Interface 4: 155-166.
9. Wu JT, Ma ES, Lee CK, Chu DK, Ho PL, et al. (2010) The infection attack rate and severity of 2009 pandemic H1N1 influenza in Hong Kong. Clin Infect Dis 51: 1184-1191.
10. Ferguson NM, Cummings DA, Cauchemez S, Fraser C, Riley S, et al. (2005) Strategies for containing an emerging influenza pandemic in Southeast Asia. Nature 437: 209-214.
11. Longini IM, Jr., Halloran ME, Nizam A, Yang Y (2004) Containing pandemic influenza with antiviral agents. Am J Epidemiol 159: 623-633.
12. Kalnay E., Kanamitsu M., Kistler R., Collins W., Deaven D., et al. (1996) The NCEP/NCAR 40-Year Re- analysis Project. . Bull Ame Meteorol Soc 77: 437-472.

Supplementary Tables

Table A. Testing rates (no. tests/no. ILI cases) for novel 2009 A/H1N1pdm influenza during the spring (Apr. 1 to May 20, 2009), summer (May 21 to Aug. 1, 2009) and fall (Aug. 2 to Dec. 31, 2009) waves by geographic region of Mexico. Average testing rates and temporal trends are indicated for each wave; significant trends are highlighted in bold. See also Figure B for spatio-temporal variations and Figure C for age patterns in testing rates.

	**Mexico**		**Central states**		**Northern states**		**Southeastern states**	
	Average testing rate % (95%CI)	Time trend, R^2 (P value)	Average testing rate % (95%CI)	Time trend, R^2 (P value)	Average testing rate % (95%CI)	Time trend, R^2 (P value)	Average testing rate % (95%CI)	Time trend, R^2 (P value)
**Spring wave**	25.5 (19.4, 32)	**68 (0.023)**	33 (26,40)	**71.3 (0.02)**	15 (11.1,18.3)	**84 (0.004)**	14 (7, 21)	**74.4 (0.012)**
**Summer wave**	32 (29.1,35)	0.001 (0.94)	40.4 (37, 44)	3.0 (0.63)	39 (31, 47)	**58.3 (0.01)**	28.3 (23.2, 33.4)	0.01 (0.80)
**Fall wave**	32 (30, 35)	38 (0.003)	42.4 (40, 45)	35 (0.005)	26 (22.1, 30))	0.01 (0.68)	27.4 (23, 32.2)	48.4 (0.001)
**All 3 waves**	31 (29,33)	25 (0.001)	32.5 (25.4, 39.6)	33.5 (0.0001)	28 (24, 32)	1.3 (0.5)	25 (22, 28)	27.3 (0.0007)

Table B. Mean estimates of the reproduction number and corresponding 95% confidence intervals for the spring, summer, and fall waves of the 2009 H1N1 influenza pandemic by geographic region. A fixed serial interval of three and four days, respectively, is assumed (delta distribution). The epidemic growth phase used to estimate the reproduction number consisted of 14 days for the spring wave (April 12 to April 25) and summer wave (May 21 to June 3) and 28 days for the fall wave in central states (August 5 to September 1) and northern states (August 8 to September 4). See Figure K for exact time periods considered as part of the epidemic growth phase.

**Pandemic wave**	**Geographic region**					
	**Central states**		**Southeastern states**		**Northern states**	
	**3-day serial interval**	**4-day serial interval**	**3-day serial interval**	**4-day serial interval**	**3-day serial interval**	**4-day serial interval**
**Spring wave**	1.98 (1.96, 2.0)	2.48 (2.44, 2.52)	-	-	-	-
**Summer wave**	-	-	1.72 (1.71, 1.73)	2.06 (2.04, 2.08)	-	-
**Fall wave**	1.24 (1.24,1.25)	1.33 (1.33, 1.34)	-	-	1.25 (1.24,1.25)	1.34 (1.34, 1.35)

Supplementary Figures

Figure A

Comparison of the age distribution of the population affiliated to the IMSS with that of the general Mexican population based on Census data. These distributions were not statistically significantly different (chi square test, P=0.18).

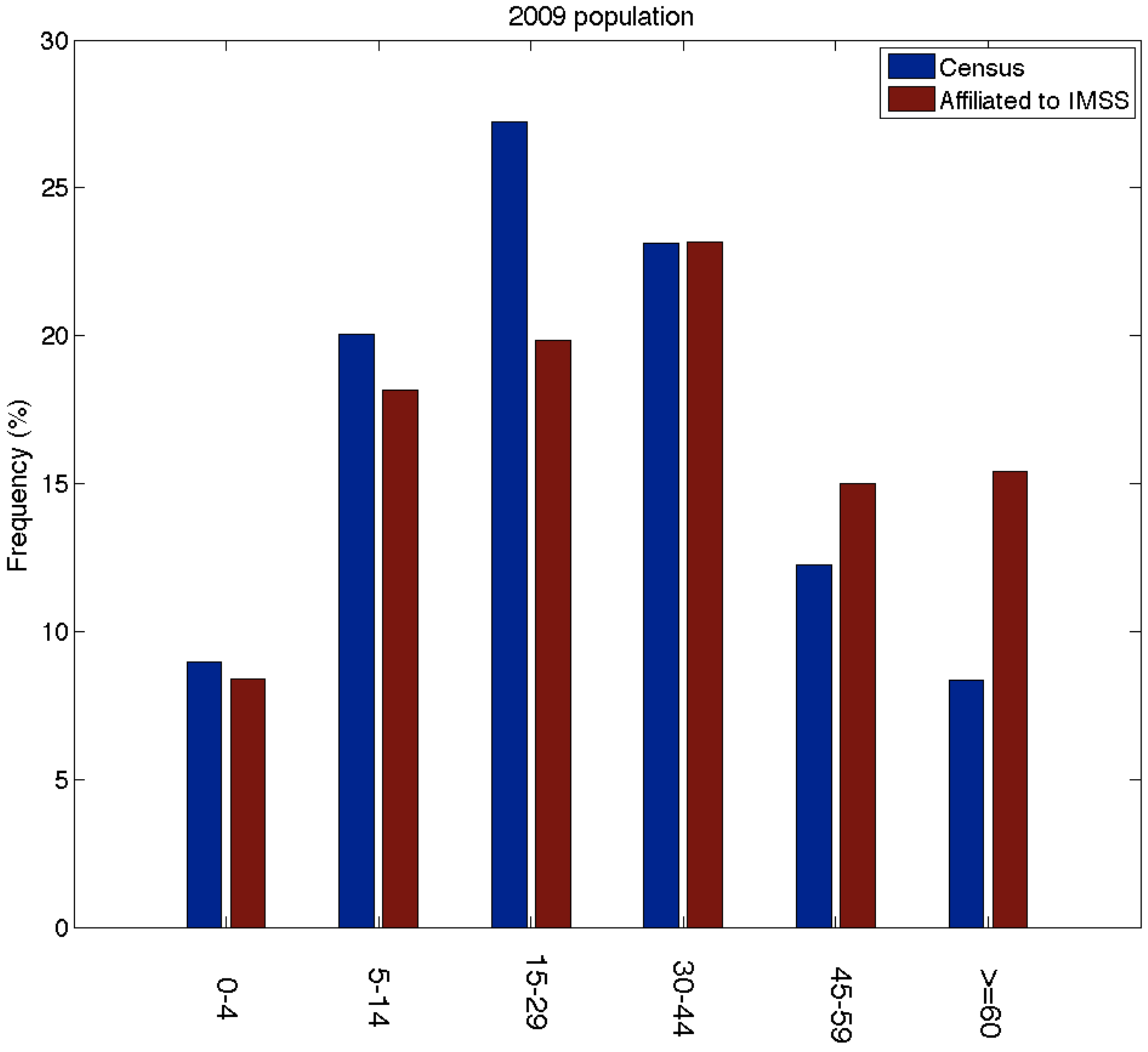

Figure B

Weekly number of ILI cases (top) and weekly variation in testing rates (no. tests/no. ILI, bottom) across northern, central, and southeastern states in Mexico, April 1 to December 31, 2009. The average testing rate during the spring wave was 25.5% (95% CI: 19.4, 32.0) and reached 32% (95% CI: 29.1, 35) during the summer wave and 32% (95% CI: 30, 35) during the fall pandemic wave (t-test for differences during the Spring and Summer/Fall, P<0.001; Table A).

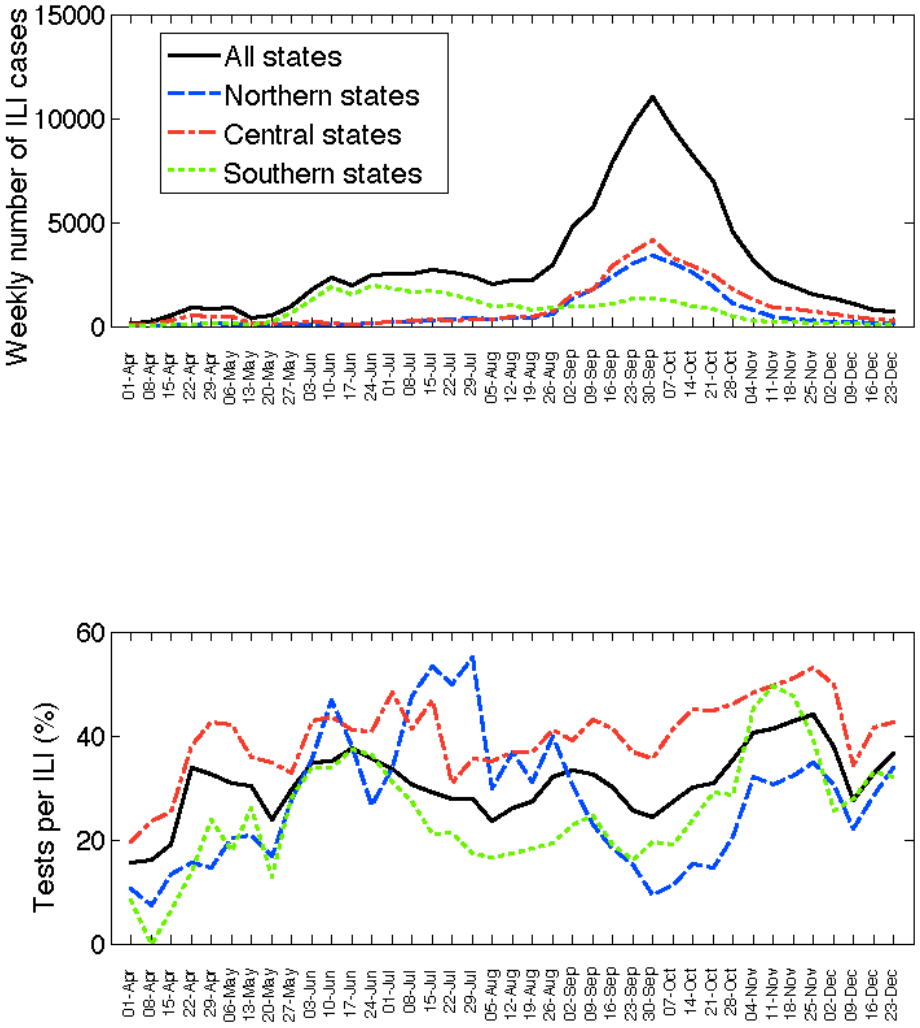

Figure C

Age-specific variation in testing rates (no. tests/no. ILI) across age groups in Mexico, April 1 to December 31, 2009. Testing rates were consistent across age groups (range: 30-33%, chi-square test; P=0.99).

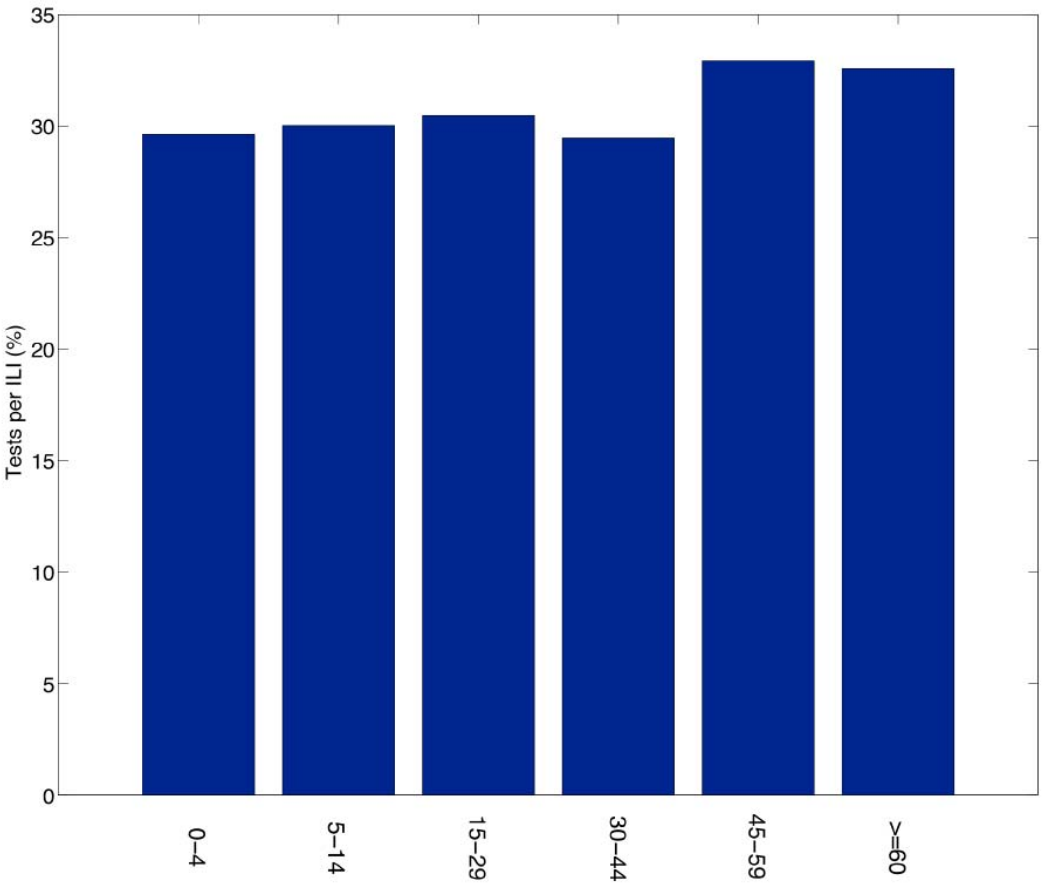

Figure D

Weekly variation in number of H1N1pdm case (top) and in percent PCR positive (no. A/H1N1pdm positive cases/no. tests, bottom) across northern, central, and southeastern states of Mexico, April 1 to December 31, 2009. By pandemic wave, the average PCR positivity was 44.3 (95% CI: 32.1, 56.4) during the spring, 78% (95% CI: 71, 85) during the summer, and 70.1% (95% CI: 63.0, 78.5) during the fall.

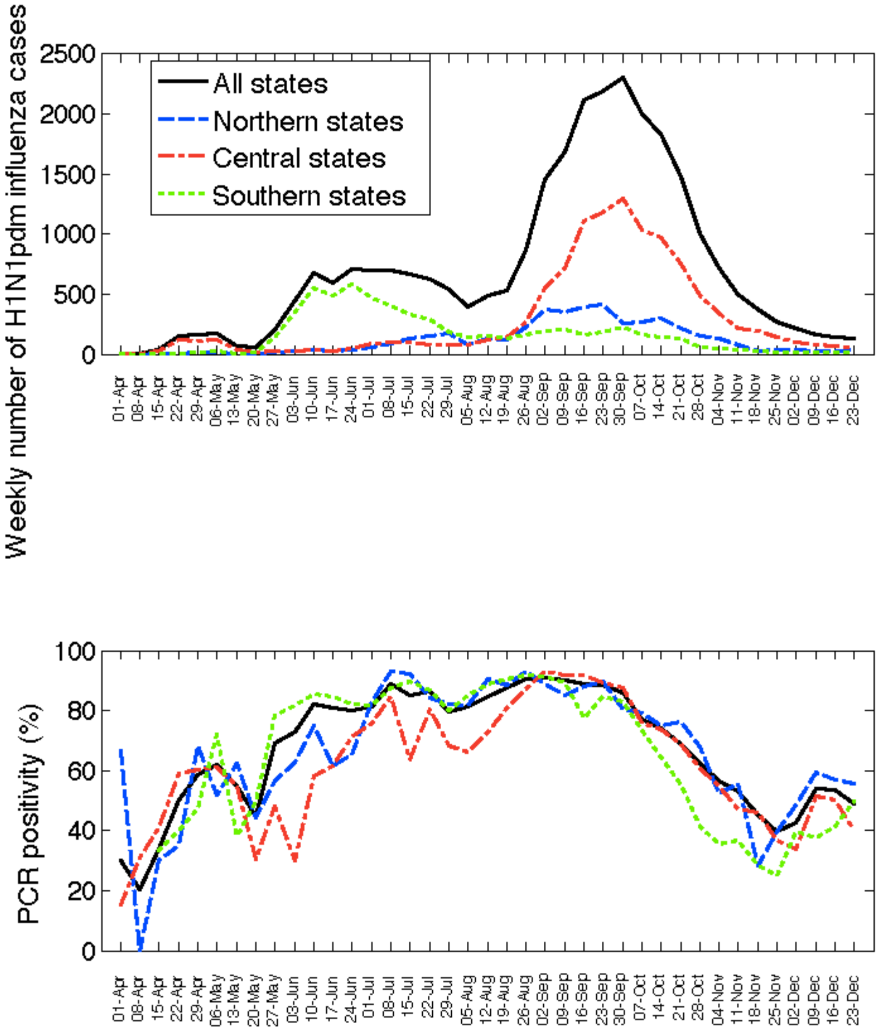

Figure E

Correlation between the incidence curves of ILI and novel H1N1 influenza cases across northern, central, and southeastern states in Mexico, April 1 to December 31, 2009.

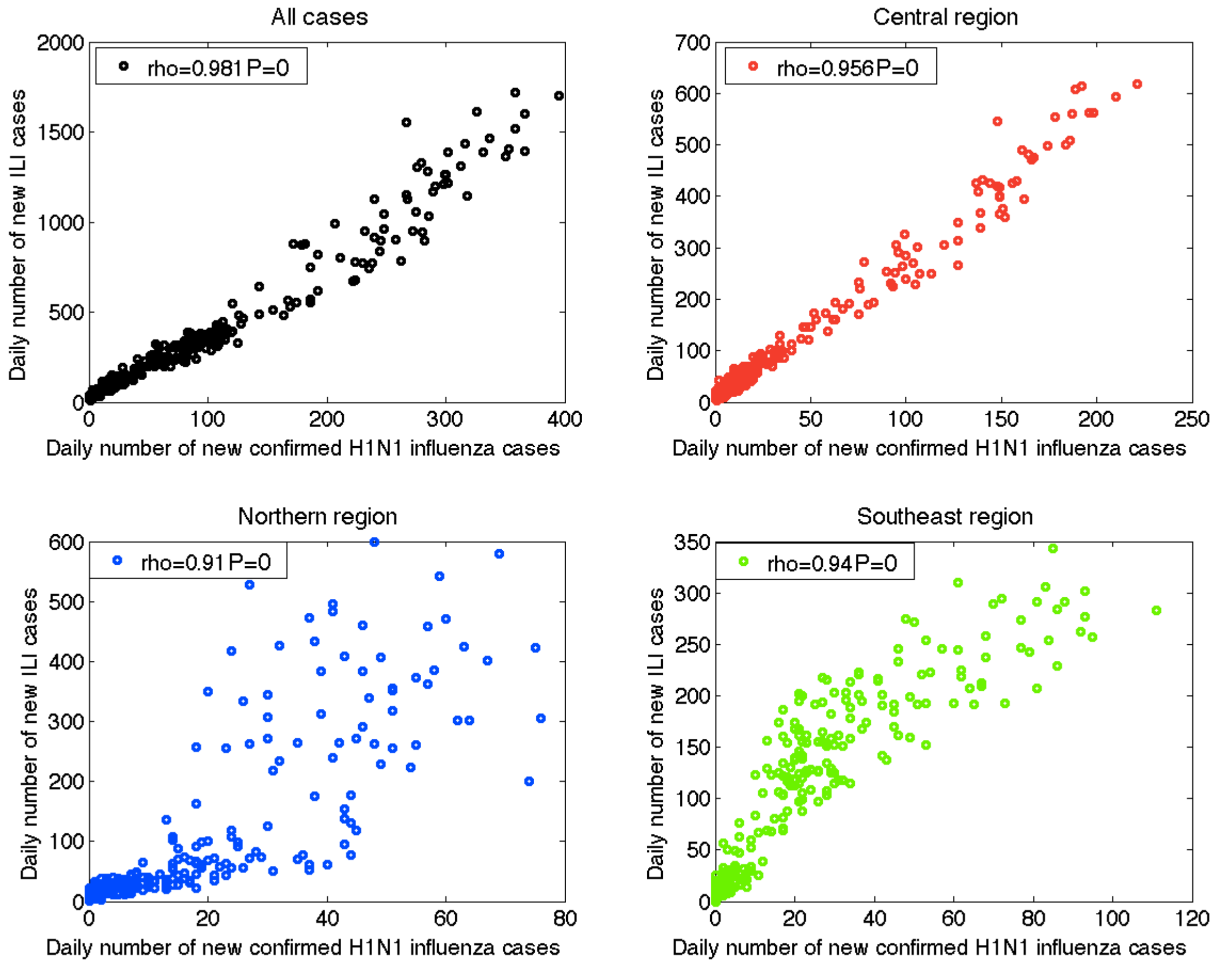

Figure F

Weekly time series of hospitalization rates (top) and mortality rates (bottom) across northern, central, and southeastern states of Mexico, April 1 to December 31, 2009.

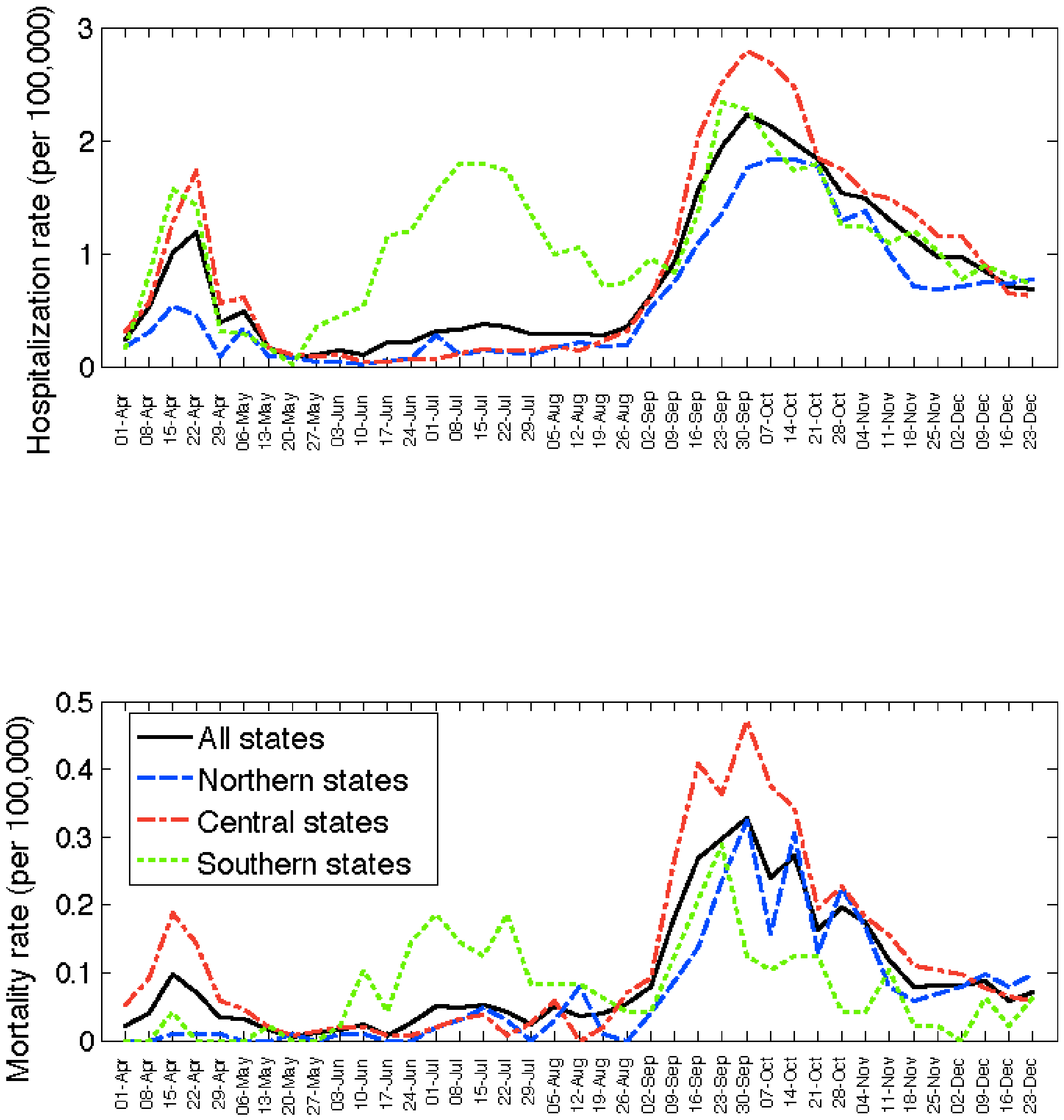

Figure G

Daily age-specific time series of influenza A/HN1pdm incidence (per 100,000) in Mexico, April 1 to December 31, 2009.

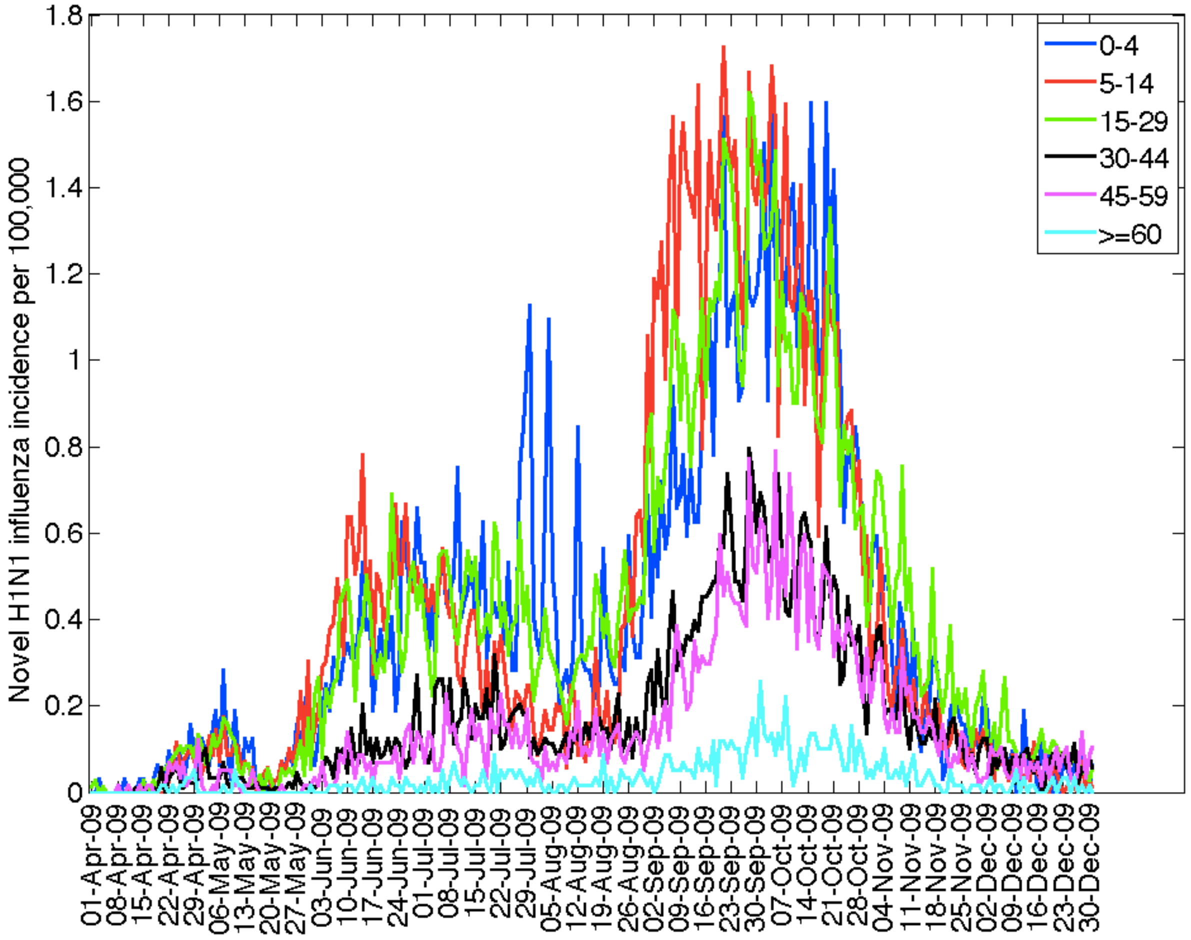

Figure H

Temporal variation in the median age of weekly H1N1pdm influenza cases throughout the 2009 pandemic period. A) The weekly number of H1N1pdm influenza cases for all 32 Mexican states and B) the weekly median age (solid line) and corresponding interquartile range (dashed lines) of H1N1pdm influenza cases during the pandemic period. There was a trend towards increasing age as the fall wave progressed (week 23-week 39; R^2=0.94, P<0.0001), with the median age reaching ~31 yrs. in December 2009.

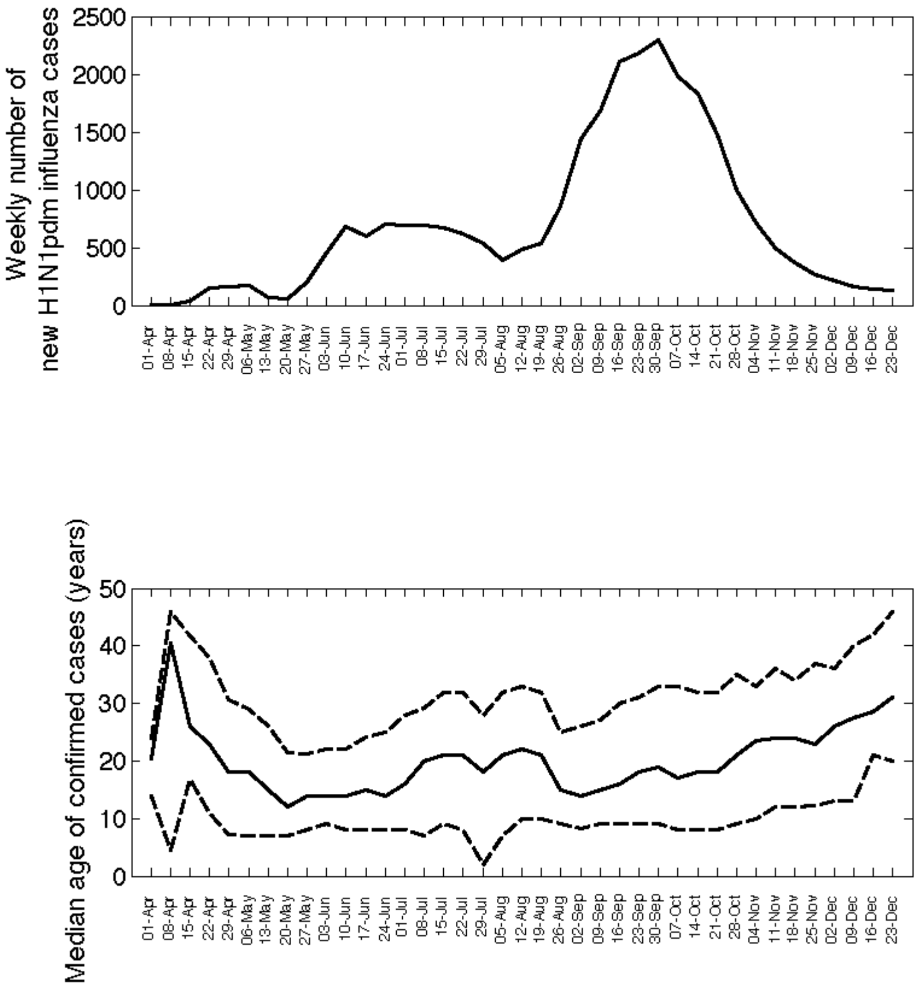

Figure I

Scaling of cumulative A/H1N1pdm morbidity rates (left) and total number of A/H1N1pdm cases (right) as a function of population size for the 32 Mexican States. Population size is negatively correlated with cumulative A/H1N1pdm morbidity rates (Spearman rho=-0.58, P<0.001) and positively correlated with total number of novel H1N1pdm influenza cases (Spearman rho=0.46, P=0.009). Right panel: The dashed blue line represent the best linear fit to the data in log-log scale. A solid black line representing a slope of one is shown as a reference to illustrate the expected relationship if morbidity rates did not vary with population size. The slope of the observed data is 'sublinear' (mean=0.46, 95%CI: 0.18-0.78), suggesting that less populous areas experienced larger epidemics. Similar patterns were evidenced in ILI data.

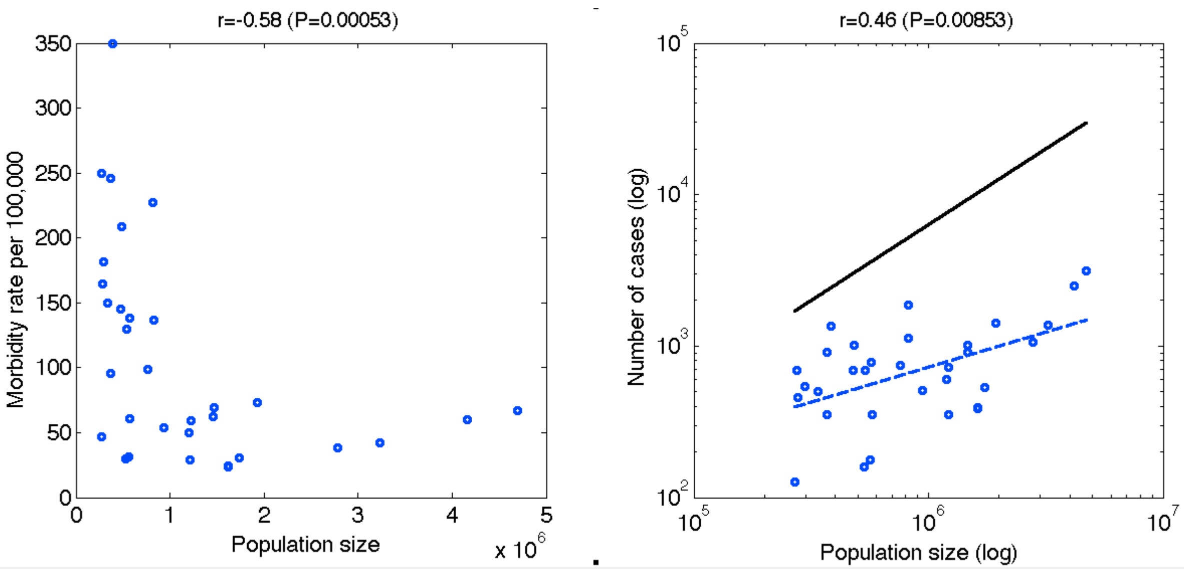

Figure J

Timing of A/H1N1pdm pandemic onset (in days) in the fall of 2009 in 32 Mexican States, as a function of population size. This suggests that larger states experienced earlier onset in the fall.

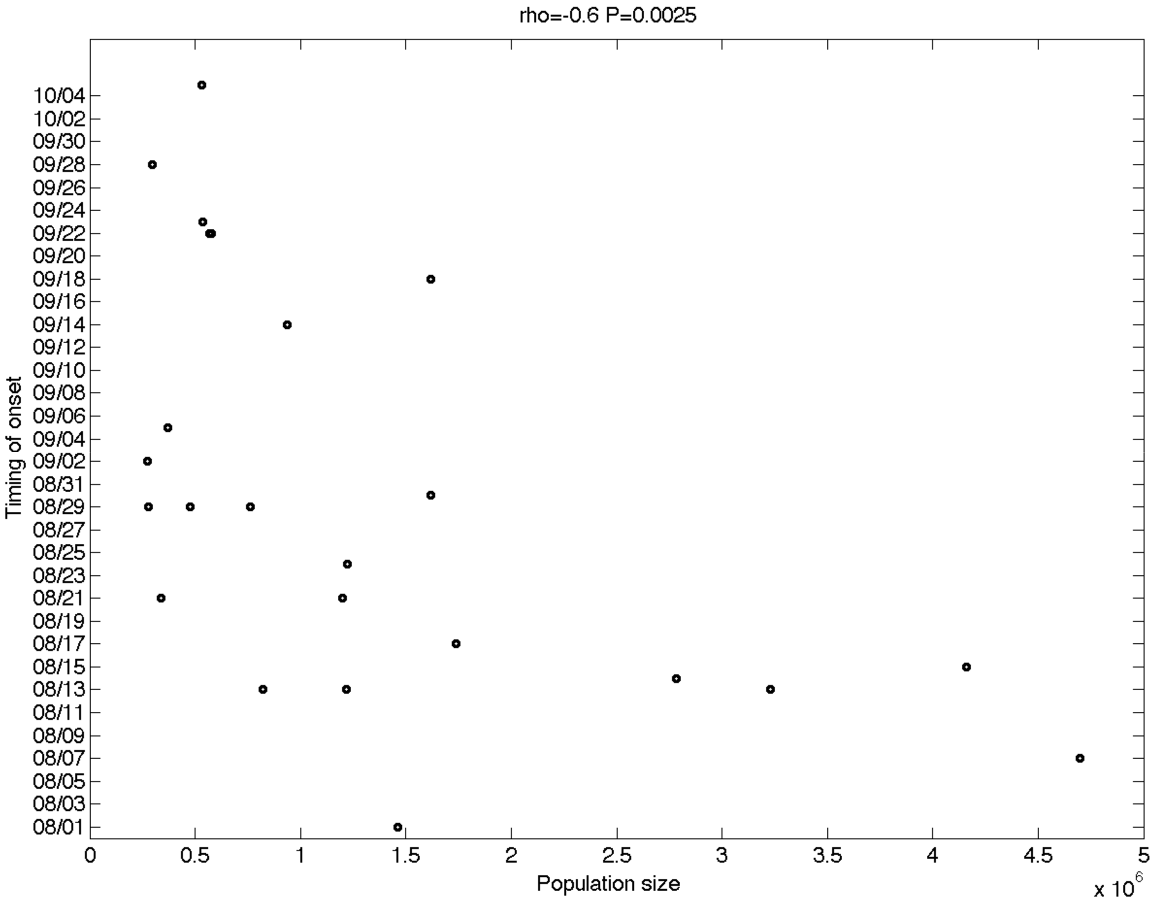

Figure K

Model fits (solid line) obtained after fitting an exponential curve to the growth phase (red circles) of the spring, summer, and fall pandemic waves in the 3 Mexican geographic regions to estimate the initial growth rate “r”. The growth phase that was selected to be representative of each of the pandemic waves consisted of 14 epidemic days for the spring and summer waves and 28 days for the fall wave.

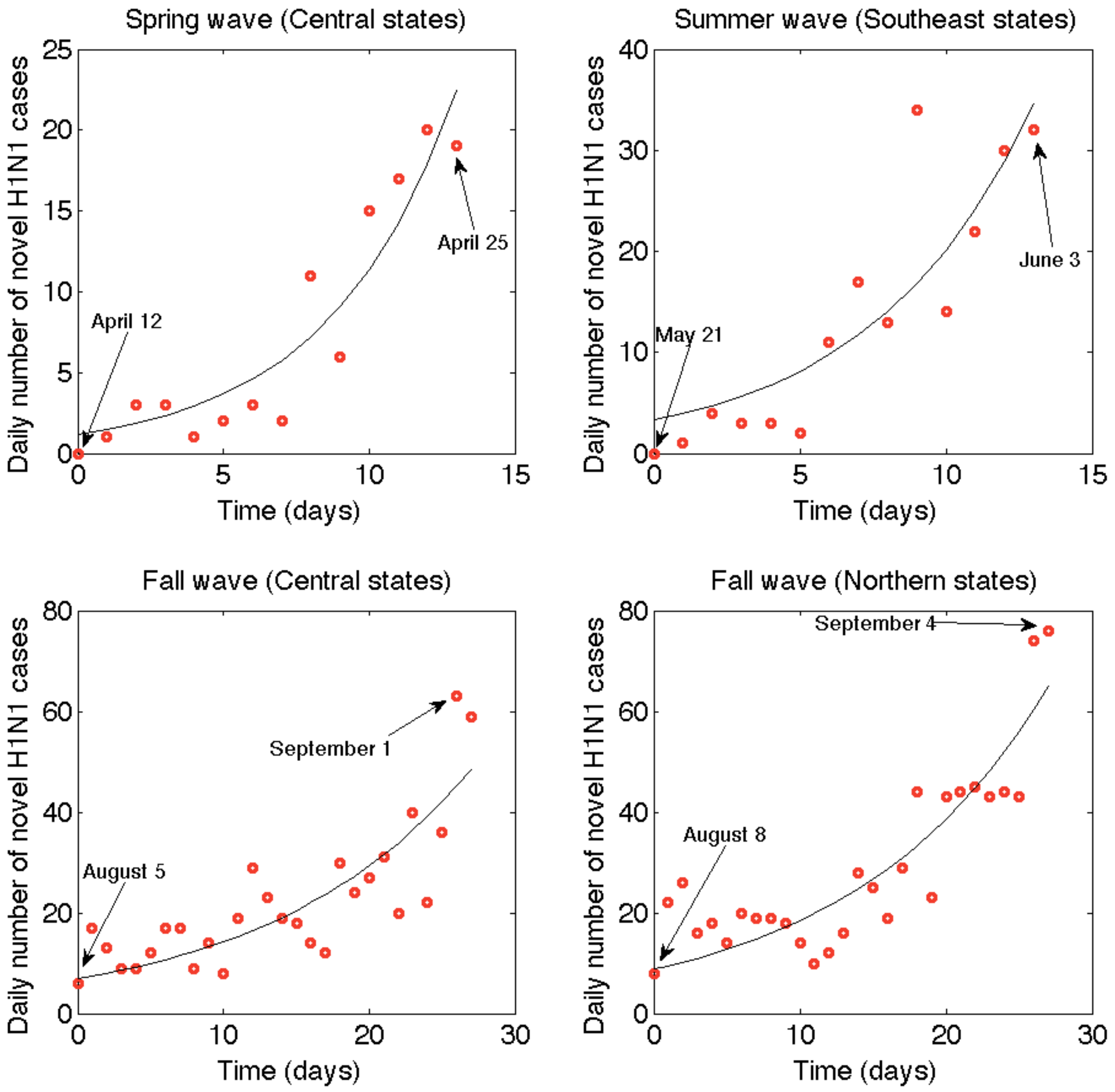

Figure L

Weekly time series of A/H1N1pdm cases (left panels) among students (5-20 y, red curve) and all other age groups (blue curve) and the corresponding weekly ratio of student to nonstudent incident cases (right panels), for five representative states of Mexico during the summer and fall pandemic waves. The grey shaded areas indicate the summer vacation period (July 03 - August 23) for elementary and secondary school students. College students retuned to classes for the fall school term on August 10th (indicated by dashed line).

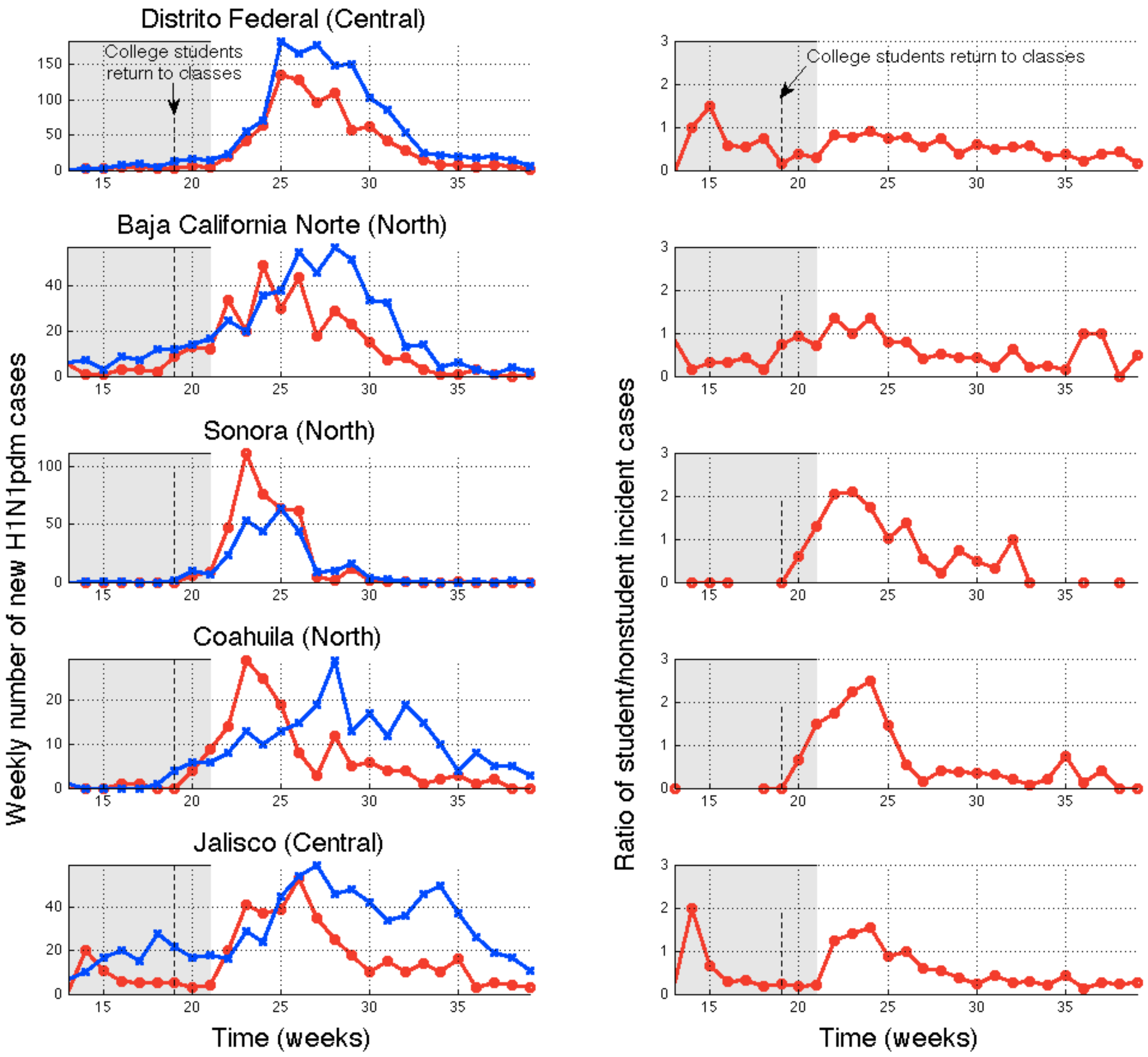

Figure M

Weekly time series of ILI cases (left panels) among students (5-20 y, red curve) and all other age groups (blue curve) and the corresponding weekly ratio of student to nonstudent incident cases (right panels), for five representative states of Mexico during the summer and fall pandemic waves. The grey shaded areas indicate the summer vacation period (July 03 - August 23) for elementary and secondary school students. College students retuned to classes for the fall school term on August 10th (indicated by dashed line).

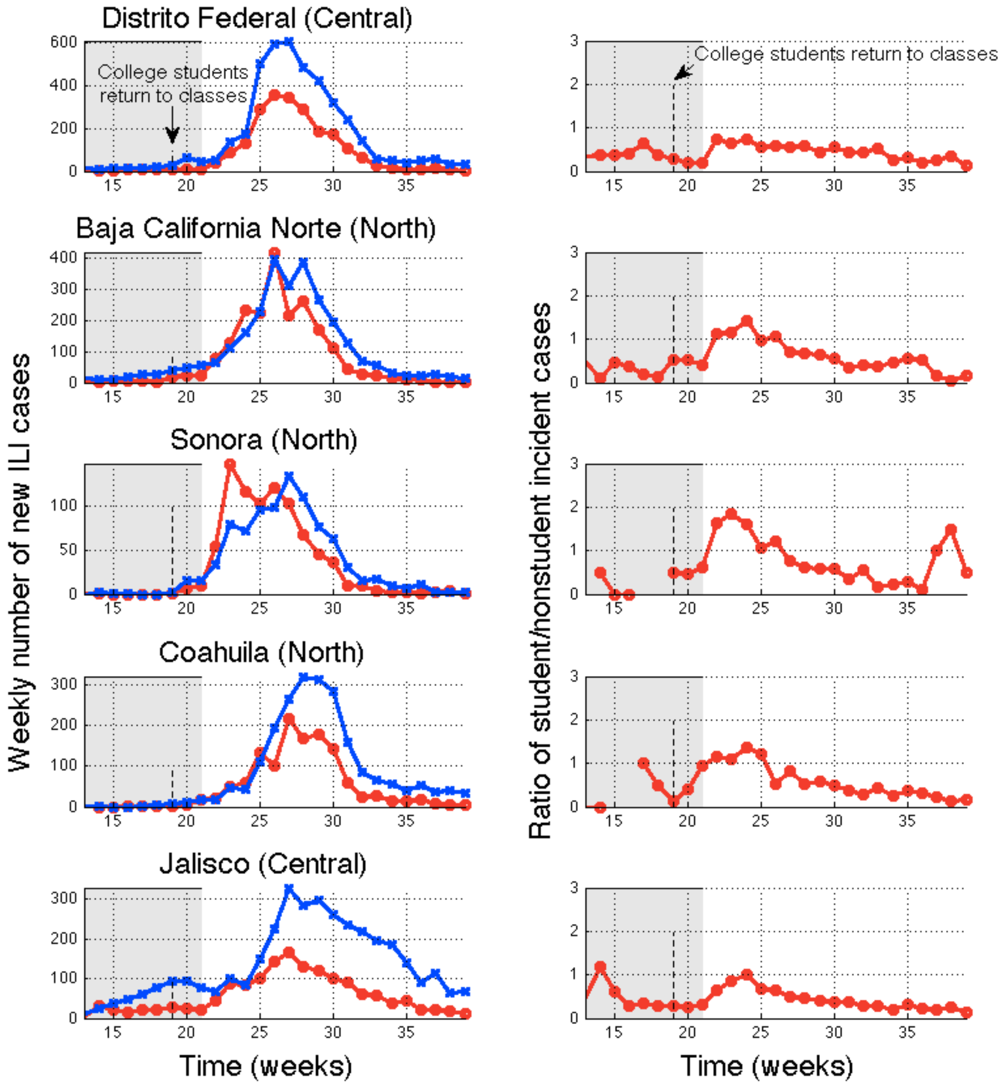

Figure N

Daily variation in number of new H1N1pdm influenza cases (top) and the average specific humidity in Mexican States (bottom) weighted by population size, April 1 to December 31, 2009.

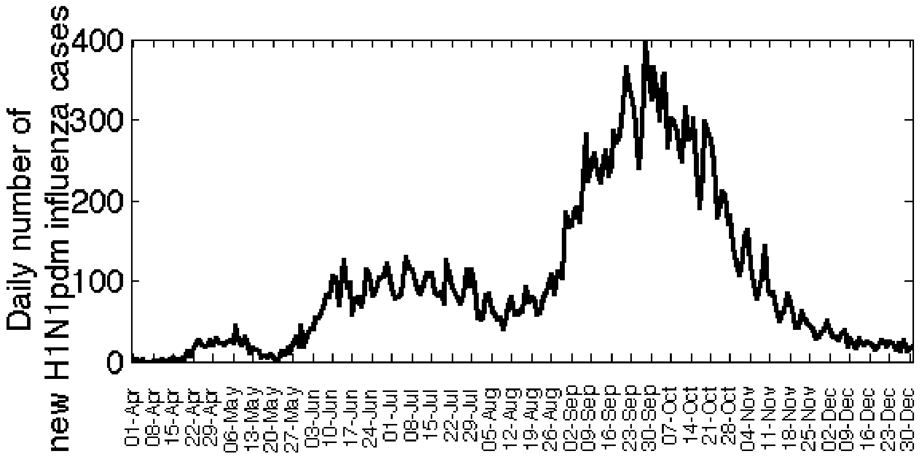

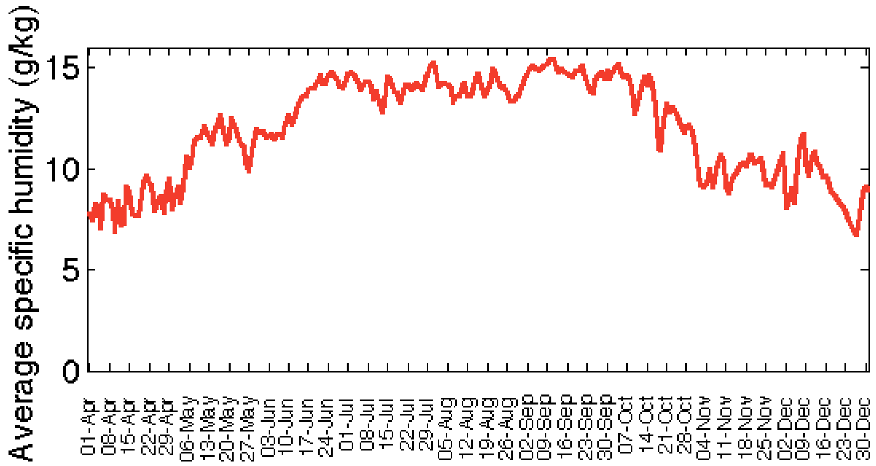

Supplement: Text S1 — Characterizing the epidemiology of the 2009 influenza A/H1N1pandemic in Mexico: Supplementary information. (0.37 MB PDF) [file pmed.1000436.s002.pdf]
